# Anti-infective Medicines Use in Children and Neonates With Pre-existing Kidney Dysfunction: A Systematic Review

**DOI:** 10.3389/fped.2022.868513

**Published:** 2022-04-26

**Authors:** Chiara Minotti, Elisa Barbieri, Denis Doni, Cristina Impieri, Carlo Giaquinto, Daniele Donà

**Affiliations:** ^1^Division of Pediatric Infectious Diseases, Department of Women's and Children's Health, University of Padova, Padova, Italy; ^2^Department of Women's and Children's Health, University of Padova, Padova, Italy

**Keywords:** kidney impairment, kidney failure, chronic kidney disease, acute kidney injury, children, neonates, anti-infective, anti-microbic

## Abstract

**Background:**

Dosing recommendations for anti-infective medicines in children with pre-existing kidney dysfunction are derived from adult pharmacokinetics studies and adjusted to kidney function. Due to neonatal/pediatric age and kidney impairment, modifications in renal clearance and drug metabolism make standard anti-infective dosing for children and neonates inappropriate, with a risk of drug toxicity or significant underdosing. The aim of this study was the systematic description of the use of anti-infective medicines in pediatric patients with pre-existing kidney impairment.

**Methods:**

A systematic review of the literature was conducted according to the Preferred Reporting Items for Systematic Reviews and Meta-Analyses (PRISMA) guidelines. The EMBASE, Medline and Cochrane databases were searched on September 21st, 2021. Studies in all languages reporting data on pre-defined outcomes (pharmacokinetics-PK, kidney function, safety and efficacy) regarding the administration of anti-infective drugs in children up to 18 years with pre-existing kidney dysfunction were included.

**Results:**

29 of 1,792 articles were eligible for inclusion. There were 13 case reports, six retrospective studies, nine prospective studies and one randomized controlled trial (RCT), reporting data on 2,168 pediatric patients. The most represented anti-infective class was glycopeptides, with seven studies on vancomycin, followed by carbapenems, with five studies, mostly on meropenem. Antivirals, aminoglycosides and antifungals counted three articles, followed by combined antibiotic therapy, cephalosporins, lipopeptides with two studies, respectively. Penicillins and polymixins counted one study each. Nine studies reported data on patients with a decreased kidney function, while 20 studies included data on kidney replacement therapy (KRT). Twenty-one studies reported data on PK. In 23 studies, clinical outcomes were reported. Clinical cure was achieved in 229/242 patients. There were four cases of underdosing, one case of overdosing and 13 reported deaths.

**Conclusion:**

This is the first systematic review providing evidence of the use of anti-infective medicines in pediatric patients with impaired kidney function or requiring KRT. Dosing size or interval adjustments in pediatric patients with kidney impairment vary according to age, critical illness status, decreased kidney function and dialysis type. Our findings underline the relevance of population PK in clinical practice and the need of developing predictive specific models for critical pediatric patients.

## Introduction

Congenital anomalies of the kidneys and urinary tract (CAKUT) are the leading cause of kidney failure in children younger than 10 years, while acquired glomerulonephritis is the leading cause in older children and adolescents. The prevalence of pediatric chronic kidney disease (CKD), defined according to Kidney Disease: Improving Global Outcomes (KDIGO) guidelines as abnormalities of kidney structure of function, present for more than three months, and classified on cause, glomerular filtration rate-GFR category and albuminuria category, is estimated as 2.7 per 1,000 children and adolescents (1, 2). Critically ill children requiring intensive care often present with a condition of acute kidney injury (AKI), described according to KDIGO guidelines as oliguria for more than 6 hours, a rise in serum creatinine level by >0.3 mg/dl in two days or by > 50% in one week, and classified by cause and stage (1).

Issues in drug dosing in children are due to a need of dose adjustment to size and developmental changes influencing drug metabolism. Dosing according to weight may lead to underdosing and often adjustments to body surface area (BSA) are preferred. Other physiological indicators of distribution and elimination, all connected to BSA, are extracellular water volume, cardiac output and renal function. Moreover, drug distribution volume is influenced by body composition, protein binding profile and metabolic clearance changes during development (3).

The main issues in this special sub-population are drug and metabolites accumulation in case of renal clearance, with possible increase of adverse reactions. Dose reductions are usually required for estimated filtration rates (eGFR) lower than 30–40 ml/min/1.73m^2^, but with potential overestimation of eGFR in small children for calculation based on creatinine. For drugs with a narrow therapeutic range, a dose reduction is a suitable option, while dosing intervals may be extended in case of administration of drugs with long plasma half-lives. Therapeutic drug monitoring is desired, considering that a reduced metabolic state increases half-life and also the time to reach steady-state levels. On the contrary, the need of kidney replacement therapy-KRT may increase the risk of sub-therapeutic drug levels. Critical illness also determines marked changes in PK, with modifications due to systemic inflammatory response and capillary leak syndrome, hypoalbuminemia and altered renal blood flow (3).

Specific subpopulations are not addressed in pediatric anti-infective drugs development, such as children and neonates with kidney dysfunction or KRT. As pointed out in a recent review, clinical evidence in children does not exist for most renal dosing recommendations in a widely used pediatric dosing handbook, with the adult recommendations from the manufacturer's label being the primary source for pediatric dosing (4). Most studies on drug dosage in patients with kidney failure are performed in adults for ethical reasons, and off-label use is frequent in pediatrics. Dosing regimens for adults with renal impairment derive from clinical and pharmacokinetics (PK) studies or population PK analysis. Adult guidelines are adapted into pediatric doses and adjusted to kidney function (3, 5).

Due to both neonatal/pediatric age and kidney impairment, renal clearance and drug metabolism modifications make standard anti-infective medicines dosing for children and neonates inappropriate, with a risk of drug toxicity or, on the other hand, significant underdosing. For antibiotics indeed, sustained sub-therapeutic drug concentrations may lead to treatment failure and contribute to further development of antimicrobial resistance (6–13). The pharmacokinetics of these medicines is rarely described for pediatric patients with pre-existing kidney injury, AKI, CKD or KRT (continuous or intermittent KRT –CKRT, hemodialysis-HD, hemodiafiltration-HDF, continuous veno-venous hemodiafiltration-CVVHD, peritoneal dialysis-PD, etc.) and recommendations are needed, as anti-infective drugs, especially antibiotics, are among the most commonly prescribed drug classes in children (14–17).

### Aim of the Study

The aim of this study was the systematic description of studies on the use of anti-infective medicines in neonates and children with pre-existing kidney injury, AKI, and KRT.

## Methods

### Study Design, Data Source, and Search Strategy

We conducted a systematic review following the Preferred Reporting Items for Systematic Reviews and Meta-Analyses (PRISMA) guidelines (18). We led a systematic search of the MEDLINE, EMBASE (1,410 retrieved titles and abstracts), and Cochrane Library databases (382 retrieved titles and abstracts), including citations from January 1946 to September 21^st^ 2021, obtained combining Medical Subject Heading (MeSH) e and free-text terms for “neonates/children” AND “anti-infective” AND “dose” AND “renal impairment.” The last search was conducted on September 21^st^ 2021. The full search strategy is available in the Supplementary Material file.

### Objectives

The primary objective of this study is the systematic description of the use of anti-infective drugs in pediatric patients with kidney dysfunction, from reduced kidney function to AKI, CKD, or on KRT. In particular, we considered PK data, kidney dysfunction and KRT if specified, dosing and duration regimen and patients' clinical outcome, if specified, defined as clinical cure, toxicity or adverse events, death.

### Inclusion Criteria

Studies in all languages were considered eligible for full-text review if they included data on neonates and children up to 18 years. Randomized controlled trials (RCTs), cohort studies, case-control studies, case series, case reports, PK studies were included.

### Exclusion Criteria

All studies on adults, systematic and narrative reviews, commentaries, editorials, book chapters, conference abstracts were excluded. Articles on children where extraction of pediatric data was not possible, and PK *ex vivo* models were also excluded.

### Study Selection and Risk of Bias Assessment

Assessment of titles, abstracts and full texts were conducted independently by three investigators (CM, CI, DeD). Discussion with a fourth reviewer (DaD) resolved disagreements regarding study selection. Clinical trials were assessed using the criteria and standard methods of the Cochrane risk of bias (RoB) tool for randomized trials (19). RoB was assessed through six criteria: risk of selection bias (random sequence generation, allocation concealment), performance bias (blinding of participants and personnel), detection bias, attribution bias (incomplete outcome data), reporting bias (selective reporting) and other bias. The risk of bias for non-randomized studies of interventions was assessed according to the Quality Assessment Tool for Observational Cohort and Cross-Sectional Studies (20). For each criterion, the included studies were classified by quality rating as good, fair, or poor by three reviewers (CM, EB, DaD), and disagreements were resolved by discussion.

### Data Collection

Data extraction was conducted using a standardized data collection form, which included information about authors, year and country of publication, study design, anti-infective drug and target, population and number of patients, kidney dysfunction/KRT, PK data, dosing regimen and clinical outcomes.

## Results

**Figure 1:**
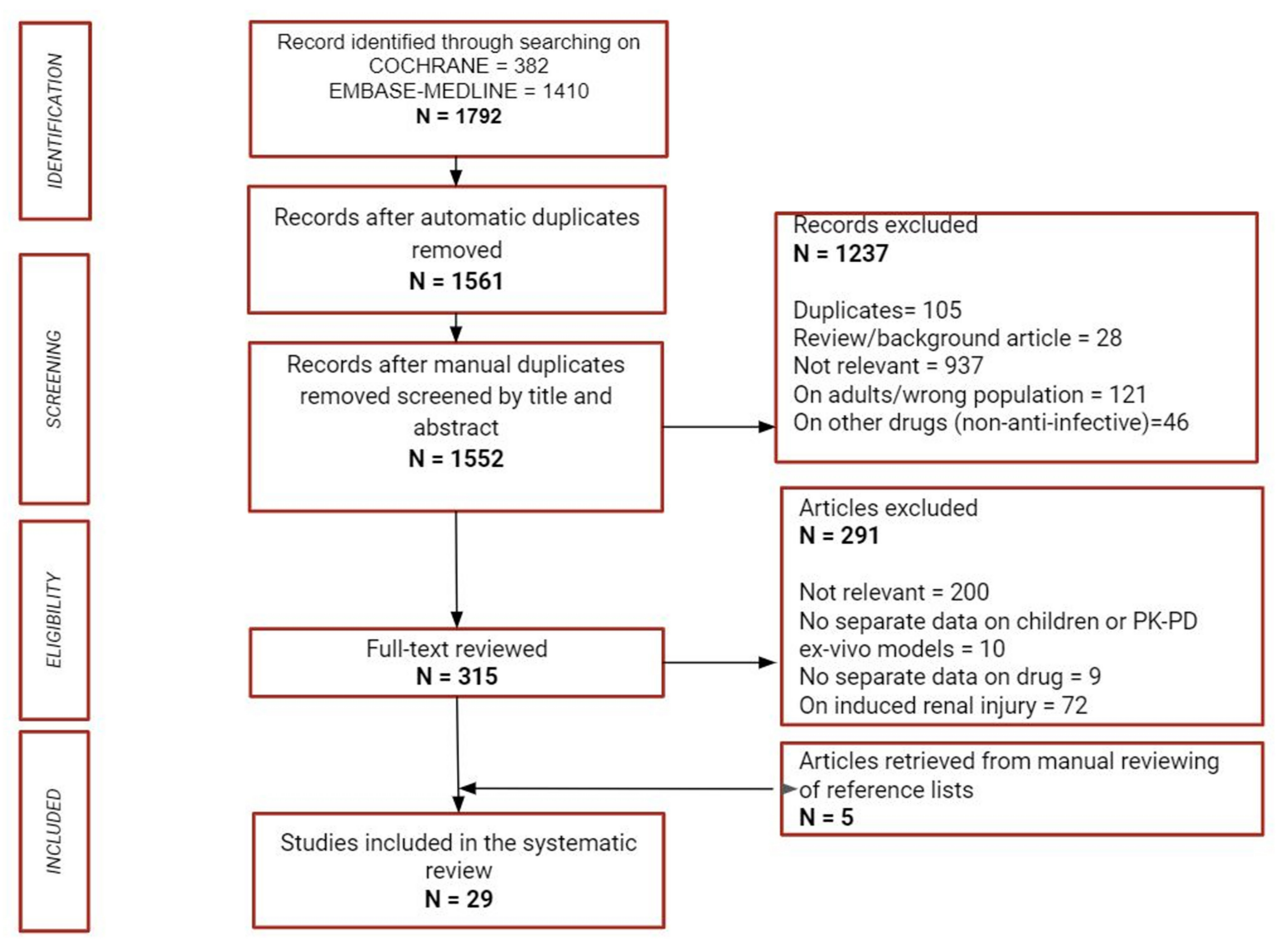
Flow chart of the study selection process.

Of 1,792 retrieved titles and abstracts, 29 were considered eligible for inclusion (21–49). A flow diagram showing the study selection process for this review according to PRISMA guidelines can be found in Figure 1. Retrieved articles with authors, country, publication year, study design and setting, kidney dysfunction characterization (CKD, AKI, KRT, if specified) and main outcomes (PK parameters, dosing regimen, clinical cure/therapy stop for toxicity/death) have been organized by anti-infective class, considering age sub-group, as summarized in Tables 1–**10**.

**Table 1:** Glycopeptides.

**Author, country, year [reference]**	**Study design and setting**	**Anti-infective drug (s)** **Target** **Dosing regimen**	**Suggested dosing regimen**	**Number of patients (*n*)**	**Kidney dysfunction/AKI/ CKD +/- KRT (type)**	**Outcome (s)**	
						**PK parameters (yes 1, no 0)**	**clinical cure 0; therapy stop for toxicity; 1 death 2; NR**
**Neonates**							
Company-Albir et al, Spain, 2019 (22)	Case report, in-patient (NICU)	IP vancomycin, PD-related peritonitis 25 mg/L continuous infusion	NR	1	AKI CKRT (PD)	0	0 (1/1)
**Children**							
Sridharan et al, Bahrain, 2019 (25)	Retrospective cohort study, in-patients (PICU) Descriptive PK	IV vancomycin Mean dose 9 mg/lg/dose; mean duration 10 days,	NR	9	Kidney dysfunction according to clearance	1 lower CL, prolonged *t*1/2, and higher AUC values	NR for subpopulation
Abid et al, USA, 2020 (26)	Case report, in-patient (no ICU)	IP vancomycin, MRSA osteomyelitis 20 mg/L continuously 14 h/day, for 2 weeks;	NR	1	CKD CKRT (PD)	0	0 (1/1)
**Children and Adolescents**							
Smit et al, USA, 2021 (27)	Retrospective study, in-patients (ICU) PK model with Bayesian adjusrment	IV vancomycin Intermittent infusion; First dose: 15 mg/kg; then:	NR	1,892	Kidney dysfunction according to Bedside Schwartz creatinine clearance (CrCl down to 8.6 mL/min/1.73 m^2^)	1	NR
		CrCL 50–90 ml/min/1.73 m^2^: 11 mg/kg every 6 h (<30 kg); 11 mg/kg every 8 h (30–70 kg); 12 mg/kg every 12 h (>70 kg)	NR				
		CrCL 30–50 ml/min/1.73 m^2^: 5 mg/kg every 6 h (<30 kg); 5 mg/kg every 8 h (30–70 kg); 6 mg/kg every 12 h (>70 kg)	NR				
		CrCL 10–30 ml/min/1.73 m^2^: 5 mg/kg every 12 h (<30 kg); 3 mg/kg every 12 h (30-70 kg); 3 mg/kg every 12 h (>70 kg)	NR				
Fitzgerald et al, USA, 20 19 (23)	Retrospective cohort study, in-patients (PICU) Therapeutic drug monitoring	IV vancomycin, empirical therapy post cardiac arrest Median initial dose 10 mg/kg (IQR 10–15); median initial dosing interval 8 h (IQR 6–12)	NR	16/43	AKI	1	NR for subpopulation (initial eGFR was lower in patients with AKI at day 5 and remained predictive of day 5 AKI)
**Neonates, Children and Adolescents**							
Cies et al, USA, 2016 (21)	Retrospective case series, in-patients (PICU) PK/PD study with clinical efficacy	IV intradialytic vancomycin Continuous infusion, 25 mg/L (range 18–35 ml/L)	NR	11/21	CKRT	1	0 (11/11)
Sridharan et al, Bahrain, 2019 (24)	Retrospective cohort study, in-patients (PICU)	IV vancomycin, empirical therapy Mean dose 13 mg/kg/dose 3 or 4 times daily mean duration 2 weeks (1–22 days),	NR	13/102	AKI	0	0 (10/13) 2 (3/13)

The most represented anti-infective class was glycopeptides, with seven retrieved studies on vancomycin, followed by carbapenems, with five studies. Antivirals, aminoglycosides and antifungals counted three articles, followed by combined antibiotic therapy, cephalosporins and lipopeptideswith two studies, respectively. Last, penicillins and polymixins counted one study each.

Regarding patients' age category, most studies were only on children (eleven, 37,9%), followed by children and adolescents (seven studies, 24.1%). Five (17.3%) studies were on neonates, four (13.8%) on adolescents and two (6.9%) included neonates, children and adolescents.

Nine (31%) studies reported data on patients with a decreased kidney function or kidney impairment, either CKD or AKI, while 20 (68.9%) studies included data on KRT (six on PD.

Twenty-one (72.4%) studies reported data on PK. Overall, data were reported on a total of 2,168 pediatric patients. In the 23 studies in which clinical outcomes were reported, clinical cure was achieved with the proposed dosing regimen in 229 of 242 patients. There were four cases of underdosing, in which the drug of choice did not achieve an acceptable intradialytic profile (patients treated with intradialytic intravenous-IV meropenem), and one case of overdosing in one patient treated with IV meropenem that had drug supratherapeutic drug levels. There were thirteen reported deaths (of which eight neonates treated with amphotericin B and two adolescents treated with colistin and fluconazole, respectively).

### Risk of Bias Assessment

The included studies mainly were case reports (13 studies, 44.8%); six (20.7%) were retrospective studies, nine (31%) prospective studies, with only one (3.54%) RCT. The overall risk of bias was high-moderate due to the study types and characteristics.

#### Risk of Bias Assessment for Clinical Trials

The risk of bias assessment for the only RCT is available in Supplementary Material (Supplementary Figure 1), showing a low risk of bias.

#### Risk of Bias Assessment for Observational Studies

The risk of bias assessment tool and results for the non-RCTs are available in Supplementary Material (Supplementary Figures 2.1, 2.2). Prospective and retrospective studies were all rated “fair” but one rated “good,” while case reports were rated “poor” because of intrinsic limitations to study types.

### Retrieved Articles

Almost 100% of the patients for which a clinical outcome was available achieved clinical cure, regardless of age, molecule type or KRT need, showing the efficacy and safety of the suggested dosing regimens. The cases of underdosing occurred in high-risk contexts of unusual administration routes (intradialytic IV administration) and overdosing with continuous infusion during CKRT, both with meropenem. The thirteen deaths mostly occurred in the most fragile patients, such as eight neonates with fungal invasive infections. One adolescent had a multidrug resistant (MDR) gram-negative sepsis from an extensive burn infection, despite colistin effectiveness in eradication, due to systemic complications. The other developed septic shock in biliar and peritoneal candidiasis during CKRT.

A good proportion of the retrieved studies reported data on PK, especially valuable when PK models for adjusted dosing schedules were proposed.

Details about the different anti-infective classes by age category are specified below.

#### Glycopeptides

All retrieved studies (five retrospective studies and two case reports) were about **vancomycin**, mainly on intermittent (five studies) but also on continuous infusion (three studies). Two articles dealt with PD, with the majority dealing with kidney impairment, not KRT (4 articles). Three articles provided PK parameters, one of them proposing a PK model (Table 1).

Indeed, among glycopeptides, vancomycin is widely used to treat invasive infections, such as meningitis, pneumonia and bloodstream infections, especially in Intensive Care Unit (ICU) settings. A septic state may significantly influence PK, with an increased volume of distribution and increased renal clearance, and consequently reduced serum drug concentrations (50, 51). Currently recommended doses are 10–15 mg/kg/dose every 6 h, with target trough concentrations of 10–20 mg/L, or an area under the curve/minimum inhibitory concentration ((AUC)/MIC) ≥ 400:1 (52–55), with a need of rapid attainment of therapeutic concentrations to improve morbidity and mortality. A recent consensus guideline from the USA reported an AUC_24_ target window of 400 mg/h/L to 600–800 mg/h/L in children to achieve optimal effects while minimizing nephrotoxicity (56).

Data from adults report a variable vancomycin clearance without univocal dosing recommendations. In addition, vancomycin displays time-dependent bactericidal activity, correlated with the time the drug concentration remains above the MIC (%t>_MIC_). Indeed, administration by continuous infusion reaches target concentrations more rapidly, reducing the risk of AKI, as demonstrated in adult studies (57–62).

##### Neonates

Company-Albir et al. reported the safe and effective use of intraperitoneal vancomycin in a Neonatal Intensive Care Unit (NICU) full-term patient with PD-related peritonitis (on continuous PD). Vancomycin was administered at a dose of 25 mg/L, without a loading dose. Serum levels at 2 and 5 days from treatment start were 8.4 and 7.4 mg/L, respectively, with clinical cure, even without reaching the recommended serum levels (22).

##### Children

A study by Sridharan et al. reported serum vancomycin concentrations from nine Pediatric Intensive Care Unit (PICU) children with kidney dysfunction receiving vancomycin at standard dosing. These patients had a significantly reduced drug clearance, a prolonged elimination half-life and area under the time – concentration curve during the dosing interval extrapolated to infinity. The recommended area under the time – concentration curve over 24 h (AUC_0−24_) of > 400 was achieved by 62.5% of the patients of this subgroup (25). This study demonstrated a good correlation between the trough concentration and the AUC_0−24_.

Abid et al. reported the safe and effective intraperitoneal vancomycin use to treat MRSA osteomyelitis in a 2-year-old girl on continuous PD (CPD) for kidney failure and with scarce peripheral IV access options (26). Vancomycin MIC was favorable. Vancomycin was administered intraperitoneally at a concentration of 20 mg/L for 14 h per day for 2 weeks. Serum levels on days 6 and 8 were adequate. The calculated vancomycin AUC/MIC ratio was adequate and exceeded the recommended ratio of 400. In the presence of peritonitis, 90% of intraperitoneal vancomycin is absorbed, compared to 50% in the absence of peritonitis. The International Society for Peritoneal Dialysis (ISPD) guidelines recommend a dose of intraperitoneal vancomycin of 1 g/L for initial treatment, followed by 25 mg/L for maintenance in children on CPD, or 30 mg/kg as a loading dose, followed by 15 mg/kg every 3–5 days in case of intermittent therapy with one daily exchange (63). Nevertheless, published data on children are sparse, with very little information on dosing in neonates.

Indeed, IV access unfeasibility is not unusual in infants and children, especially when the vessels need to be preserved for HD fistulae or when the risks of placing an IV access outweigh the benefits. In these cases, drug dosing and monitoring require special attention. When vancomycin is administered by continuous infusion, the AUC is calculated by multiplying the steady-state serum concentration by a factor of 24 (h). The same approach was used in the case reported by Abid et al. Intraperitoneal vancomycin exposure was considered comparable to IV administration, given that no vancomycin was eliminated over the remaining 10 h per day without PD, in a patient with kidney failure since birth and with no residual kidney function. This approach may be considered in exceptional cases, bearing in mind the tendency of a prolonged time to steady-state in dialysis patients.

##### Children and Adolescents

Smit et al. provided a dosing adaptation guideline for IV vancomycin in a wide population of 1,892 children and adolescents, including overweight, obese individuals with an impaired renal function (patients on KRT were excluded), with available vancomycin concentrations after at least two IV administrations (27). From these data, they developed a two-compartment model with inter-individual variability on clearance and peripheral volume of distribution, with a final proposed dosing guideline for intermittent IV administration, according to both kidney function (measured with bedside Schwartz formula) and body weight (doses and administration intervals are fully reported in Table 1). The aim was reaching an AUC_20_ of 400–700 mg/h/L at day 3 after treatment start. Already in the first 24 h, target AUCs were reached in all individuals with impaired renal function. Overlapping results were obtained, adapting the dosing guideline to a continuous infusion regimen (15 mg/kg as a loading dose, followed after 3 h by the proposed daily dose as 24-h infusion).

In their retrospective cohort, Fitzgerald et al. reported the therapeutic drug monitoring of IV vancomycin in 16/43 pediatric patients with AKI after cardiac arrest. The median initial vancomycin dose was 10 (IQR 10–15) mg/Kg, administered at a median interval of 8 (IQR 6–12) h. The range of initial dosing intervals was wide in patients with AKI. All children underwent vancomycin concentration measurement within 3 days from the first dose, with higher first drug concentrations in patients with AKI, indifferently below or over 1 year of age (median 16 vs 7 mg/L, P=0.003). The initial eGFR was a significant predictor of AKI on day five (23). In this special population, decreased drug clearance was evident early. However, early concentration monitoring was not performed on all patients with a low initial eGFR, indicating the need for an earlier therapeutic drug monitoring in high-risk populations with renal impairment (23).

Special consideration should be made to the recent study by Smit et al. (27). PK studies on vancomycin in the pediatric obese population are limited, especially in children and adolescents with impaired renal function. The authors demonstrated that drug clearance was well predicted by combining total body weight and renal function calculated with bedside Schwartz equation, which is normalized for body surface area, and suggested dose adjustments not on trough concentrations alone, but with Bayesian forecasting, based on PK models, such as the proposed one. These data indicate that obese children may present some hyperfiltration, though without translating into increased vancomycin clearance. With the proposed dosing, effective and safe exposures at day 3 (AUC_day3_ of 400–700 mg/h/L) and in the first 24 h are expected throughout the pediatric population over 1 year of age. The current suggested doses [IDSA and British National Formulary for children (64)], as compared to the proposed dosing regimen, resulted instead in higher exposures (AUC_day3_ >700 mg/h/L) in obese pediatric patients with reduced renal function.

##### Neonates, Children and Adolescents

Cies et al. described the use of continuous infusion vancomycin in the dialysate fluid in 11 ICU patients requiring CKRT (21). The median administered dose was 25 mg/L, with a mean plateau level of 22.8 ± 3.3 mg/L. All children were administered a loading dose before the CKRT started. When vancomycin was started after CKRT, a single loading dose of 15–20 mg/kg was given intravenously over 60 min and afterwards added continuously into the CKRT solution, dialysate and/or replacement fluid depending on hemodialysis modality. Serum levels were monitored. If the first plateau level was within range, daily plateau levels were obtained for the duration that the vancomycin was mixed in the CKRT solution(s). If the first vancomycin plateau level was out of range, the concentration in the CKRT solution(s) was adjusted. Ten out of 11 patients reached therapeutic levels within 8 h. A serum plateau level higher than 15 mg/L was reached in all patients (target 15–30 mg/L), regardless of CKRT modality, dose and flow rate. No adverse events were reported.

Data about vancomycin use in CKRT are scarce and not specific for the pediatric population. Adult data have identified factors influencing PK, such as inotropes/vasopressors, extrarenal drug clearance, CKRT intensity and circuitry, as well as albumin concentration (60, 61). Studies demonstrated an increase of drug clearance by at least 30% in changing from hemofiltration to hemodiafiltration and that ultrafiltration rates are a limiting factor for drug concentrations (62). The study by Cies et al. pushes forward the already reported practice of adding antibiotics to dialysate solutions, common in PD. Regarding its application to the pediatric population, adding vancomycin to CKRT solution has the advantage of minimizing the chance of high serum concentrations and potential toxicities, ensuring drug delivery only while receiving CKRT. Moreover, the vancomycin serum plateau level should correspond to the drug concentration in CKRT solution(s), regardless of the membrane, dialysis modality or intensity. This may also result in a lower drug consumption per day and eventually cost saving (21).

Sridharan et al. published a retrospective study on the use of empirical IV vancomycin in 13 PICU patients with renal dysfunction due to AKI, receiving standard doses (Table 1) (24). Vancomycin was dosed and resulted in the recommended range only in 22% of patients with renal dysfunction (median concentration of 7.4 mg/l). Moreover, concomitant use of other potentially nephrotoxic medicines, such as meropenem, gentamicin, piperacillin, and furosemide, was significantly more common amongst children with AKI than others. Ten patients survived and three died.

Recommended vancomycin dosing regimens may prove ineffective in reaching therapeutic trough levels in critically-ill children with renal dysfunction. This is because sub-therapeutic levels imply underdosing, while drug accumulation during AKI may lead to toxicity, with a consequent higher risk of mortality. Moreover, in ICU settings, the concomitant use of other nephrotoxic anti-infective agents or different medicines is not uncommon. The authors mention the possibility of estimating AUC_24_ and titrating vancomycin levels by a Bayesian approach using validated softwares from population-specific pharmacokinetic parameters, but with the limitation of a lack of supporting cost-effectiveness studies.

#### Carbapenems

Of the five retrieved studies on carbapenems, four were on meropenem, of which two prospective studies on children and adolescents, one dealing with HD, and two case reports, respectively on a child and a newborn, both on HD. Three studies reported data on continuous infusion. One prospective study was on panipenem in adolescents on HD. All provided PK data (Table 2).

**Table 2:** Carbapenems.

**Author, country, year [reference]**	**Study design and setting**	**Anti-infective drug (s)** **Target** **Dosing regimen**	**Suggested dosing regimen**	**Number of patients (*n*)**	**Kidney dysfunction/AKI/ CKD +/- KRT (type)**	**Outcome (s)**	
						**PK parameters** **(yes 1, no 0)**	**clinical cure 0 therapy stop for toxicity 1 death 2 NR (*n*)**
**Neonates**							
Cies et al, USA, 2016 (30)	Case report, in-patient (PICU) Descriptive PK	IV meropenem, *P. aeruginosa* sepsis Bolus 40 mg/kg, then continuous infusion 10 mg/kg/h;	NR	1	CKRT (CVVHDF)	1	0 (1/1)
**Children**							
Alqaqaa et al, USA, 2016 (29)	Case report, in-patient (PICU) Descriptive PK	IV meropenem, *E. coli* septic shock continuous infusion 10 mg/kg/h	NR	1	CKRT (CVVHD)	1 V_d_: 0.026 L/kg CL: 0.051 mL/kg/min	1 (1/1) For supratherapeutic levels shift to intermittent infusion 20 mg/kg q12 h.
**Children and Adolescents**							
Goldstein et al, USA, 2001 (28)	Prospective study, in-patients (no ICU) PK model with Bayesian adjusrment	IV meropenem, central-line infection by *P.aeruginosa* single dose of 20 mg/kg (max 500 mg) before and after two separate HD treatments.	Simulated doses for pharmacodynamic target: 25 mg/kg/day or an alternate daily dose of 40 mg/kg	7	ESKD (HD)	1	0 (3/7) (4/7): dose insufficient to reach interdialytic pharmacodynamics profile of 70% duration with a drug concentration > 4 mg/L.
Rapp et al, France, 2020 (31)	Prospective study in-patients (PICU) PK model development based on Monte Carlo simulations	IV meropenem, empirical or documented infection Administered: 20 mg/kg q8 h as a 20-min infusion, 40 mg/kg q8 h as a 20-min infusion, 20 mg/kg q8 h as a 3-h infusion, 60 mg/kg per day as a continuous infusion, and 120 mg/kg per day as a continuous infusion, duration 7.5 days (range 1–28).		14/40	Kidney dysfunction with/without CKRT	1	NR
			MIC <2 mg/L: intermittent infusion 60 mg/kg/day MIC >4 mg/L: continuous infusion 60 mg/kg/day		3/14 Kidney dysfunction without CKRT		
			MIC <2 mg/L: intermittent infusion 60 mg/kg/day MIC >4 mg/L: continuous infusion 60 mg/kg/day		11/14 Kidney dysfunction with CKRT		
**Adolescents**							
Hayakawa et al, Japan, 2006 (32)	Prospective study, in-patients (ICU) Descriptive PK	IV panipenem/beta mipron, sepsis in ALL 1 g x3/day	NR	1/4	CKRT	1 total clearance 135 ml/min clearance by CKRT 33.8 ml/min	NR

Not surprisingly, most of the retrieved studies focused on the widely-used **meropenem**, a broad-spectrum antibiotic of the carbapenem class of beta-lactams. Its main activity is against most gram-positive and gram-negative bacteria, including *P. aeruginosa*, in empirical and targeted therapy for severe infections by multi-drug resistant germs. Its PK has been described in healthy adults and children and adults with renal impairment, but not in children with renal impairment (65–70). Its efficacy is maximal for concentrations one to four times above the MIC throughout the dosing interval (71, 72). However, critically ill pediatric septic patients are prone to wide fluid balance variations and changes in blood circulation, with effects on PK parameters like the volume of distribution and clearance. Moreover, impaired kidney function and CKRT affect meropenem elimination, with a risk of suboptimal drug exposure with the usual intermittent dosing (31). The standard IV dose in children is 20 mg/kg/dose every 8 to 12 h.

##### Neonates

Cies et al. described a case of a full-term newborn that presented complications after adenovirus infection, with multi-organ failure, disseminated intravascular coagulation and eventually *P. aeruginosa* sepsis/septic shock (30). She required CKTT with hemodiafiltration and then ECMO and was treated with meropenem, first 40 mg/kg in a single bolus, followed by continuous infusion at a dose of 10 mg/kg/h (meropenem MIC=0.25 mg/L.) After 3 days, a meropenem serum concentration of 21 mg/L was obtained (clearance of 7.9 mL/kg/min), with a 100% *f* T>MIC. This regimen provided a 100% target attainment for serum concentrations above the MIC for ≥40% of the dosing interval and cultures sterilization (30). Indeed therapeutic drug monitoring is critical, and individualized, PK-based regimens may help reach optimal efficacy in these patients.

##### Children

Alqaqaa et al. reported the case of a 4-year-old boy with a recent diagnosis of leukemia in status post-induction chemotherapy who presented septic shock due to *E. coli*, requiring extracorporeal membrane oxygenation (ECMO) and CKRT (29). Meropenem was at first administered at a dose of 40 mg/kg intravenously every 8 h, together with amikacin and vancomycin. The patient presented progressive renal failure and eventually required CVVHD, so meropenem was shifted to continuous infusion at a dose of 10 mg/kg/h. Serum drug concentrations were monitored daily, registering a rise from 718 mg/L on day three (before continuous infusion) to 3,553 mg/L on the seventh day. The continuous infusion was stopped for supratherapeutic levels, and intermittent administration (20 mg/kg every 12 h) was started 12 h later, with subsequent falling trough levels. The reported volume of distribution (Vd) was 0.026 L/kg with a clearance of 0.051 mL/kg/min.

##### Children and Adolescents

Goldstein et al. and colleagues studied meropenem dosing in children with ESKD. They reported easy clearance by hemodialysis, in line with studies on adults (28). Also, the median drug half-life between dialytic sessions was similar to what was reported for adults with ESKD (73). A single dose of 20 mg/kg (maximum 500 mg) was administered before and after two separate hemodialysis (HD) treatments in seven children and adolescents (six male and one female) with *P. aeruginosa* central-line infections (28). The drug was well tolerated without adverse effects. Meropenem reduction percentage was well correlated with increasing urea reduction percentage (average increase in meropenem clearance of 295%). The median drug half-life was 7.3 h off dialysis. The 20 mg/kg dose was insufficient to ensure an acceptable interdialytic pharmacodynamics profile of 70% duration with a drug concentration > 4 mg/L (the MIC_90_ for *P. aeruginosa*) in four of the seven patients. Simulated doses able to guarantee an acceptable pharmacodynamics profile, achieving suitable peak concentrations, were 25 mg/kg/day or an alternate daily dose of 40 mg/kg.

The chosen single dose of 20 mg/kg followed data from the literature reporting no dose-dependent effects on PK in children receiving doses from 10 to 40 mg/kg. However, such a dose was inadequate to maintain bactericidal concentrations between dialysis sessions. According to the model of dosing simulation, a dose of 40 mg/kg every other day could ensure concentrations above 4 mg/L in children with ESKD, by administering the drug after every dialytic session. Therefore, the suggested regimen for children on dialysis in an ICU setting would be 25 mg/kg/day after dialysis treatment, hypothesizing daily sessions (28).

Rapp and colleagues developed a PK model for meropenem, confirming that CKRT is an influential parameter on clearance for easy drug diffusion through the dialysate membrane. They described the development of a meropenem PK model in 40 critically-ill PICU children, of which 14 with renal impairment and 11 on CKRT (31). Data were fitted with a two-compartment model with first-order elimination (PK parameters: individual clearance-CL, central volume of distribution, individual inter-compartment clearance-Q and peripheral volume). As eGFR had a significant effect on individual clearance, a final CL covariate submodel for children with CRTT was developed. Monte Carlo simulations were used to determine the most suitable dosing regimen for patients with severe kidney failure with CKRT or without CKRT, defined as the probability of target attainment as 50% of the time that free drug remains above the MIC over a 24-h period (fT>MIC) and 100% fT> MIC, at steady state. Meropenem dosing regimens are listed in Table 2. For each dosing regimen, thethreshold for nephrotoxicity (44 ml/L) was crossed for patients with severe renal failure for doses of 120 mg/kg/day. The suggested regimen for children with renal failure, regardless of the need of CKRT, was intermittent infusion 60 mg/kg/day for MIC < 2 mg/L or continuous infusion 60 mg/kg/day for MIC > 4 mg/L.

The suggested dose of 60 mg/kg/day, either by intermittent or continuous administration, is consistent with pediatric recommendations. However, further studies on children on CKRT only, focusing on the effects of dialysate/filtration flow rate on elimination, are needed. For bacteria with a high MIC of > 4 mg/L, continuous infusion appeared to be the best regimen to achieve 50% or 100% *f* T>_MIC_ in children on CKRT, as well as for those with normal or augmented renal clearance. For germs with lower MIC, intermittent administration was appropriate in children with renal failure, on CKRT and with normal renal function alike, but not for children with an augmented renal clearance. Therapeutic drug monitoring is especially necessary for these vulnerable populations, considering that doses of 120 mg/kg/day, either on intermittent or continuous infusion, may lead to accumulation and possible toxicity.

##### Adolescents

One of the included studies focused on panipenem/betamipron (PAPM), a carbapenem active against Gram-negative and Gram-positive, aerobic and anaerobic bacteria, mainly eliminated by the kidneys (32). It is administered with beta-mipron (BP), an organic anion tubular transport inhibitor inhibiting the active transport of panipenem in the renal cortex and thus reducing its nephrotoxic potential. For panipenem, it is desirable to maintain a concentration above the MIC through the dosing interval. The recommended dose for adults with no renal impairment is 0.5 g every 12 h, increasing to 1 g every 12 h, with no reported data on children. Dialysate and ultrafiltrate flow on CKRT are expected to influence its PK (74–76).

Hayakawa et al. provided data on the PK and most suitable dosing regimen of PAPM in four critically ill patients on CKRT, of which one 16-year-old female (32). They developed an *in vitro* model to estimate PK, offering a predictive formula to determine the appropriate regimens during CKRT, calculating panipenem total clearance as the sum of clearance dependent on the body together with CKRT. Most importantly, the influence of CKRT-dependent clearance on total clearance increases when the high-volume replacement CKRT is done in case of severe AKI. In PK simulation, the doses and intervals for administration were adjusted to maintain the breakpoint MIC of 4 mg/L throughout the dosing intervals. Total drug clearance during CKRT was estimated based on dialysate flows, ultrafiltrate flows and renal function of patients. PK values of the drug were measured in the four patients. The adolescent patient had a 24-h creatinine clearance (CrCL) of 32 ml/min and received 1 g x3/day of PAPM. Reported PK values during CKRT were: AUC 124 mg/h/ml, half-life 4.6 h, steady-state volume 0.84 l/kg, total clearance 135 ml/min and clearance by CKRT 33.8 ml/min, with almost equal predictive clearance values. The appropriate dosing regimens to maintain an adequate plasma concentration level throughout the dosing intervals were established through predictive formulas applied to various renal functions during CKRT.

Last, it is worth reporting the experience of Ye and colleagues in establishing an *ex-vivo*, physiologically-based PK (PBPK) simulation model for prediction of the PK of **ertapenem** in pediatric patients with renal impairment and PD evaluation after validation of adult data (76). Ertapenem concentrations were simulated in children with kidney impairment after an intravenous administration of 15 mg/kg, chosen for healthy individuals with a target of achieving 40%T>MIC. Despite no significant changes in maximum/peak serum concentration (C_max_), the AUC value was 1.42-fold higher in mild kidney impairment, 1.84-fold higher in moderate renal impairment and 3.52-fold higher in ESRD than in healthy children. Based on these changes, the dose was adjusted to 13 mg/kg bis-in-die (bid) in mild renal impairment, 9 mg/kg bid in moderate renal impairment, 6 mg/kg bid in severe renal impairment, and 5 mg/kg bid in ESRD. %T> MIC was evaluated, with a probability of reaching 40%T>MIC (MIC ≤ 4 mg/L) of nearly 100%. As there is no data on ertapenem for pediatric patients with renal impairment, a PBPK model represents an important tool for incorporating changes in this special population.

#### Aminoglycosides

The three prospective studies on aminoglycosides were about gentamicin (two studies, on children/adolescents and children respectively) and amikacin (one study on children). None of them dealt with any dialysis modality, while all reported PK data (Table 3).

**Table 3:** Aminoglycosides.

**Author, country, year [reference]**	**Study design and setting**	**Anti-infective drug (s)** **Target** **Dosing regimen**	**Suggested dosing regimen**	**Number of patients (*n*)**	**Kidney dysfunction/AKI/ CKD +/- KRT (type)**	**Outcome (s)**	
						**PK parameters** **(yes 1, no 0)**	**clinical cure 0 therapy stop for toxicity 1 death 2 NR (*n*)**
**Children**							
Sirinavin et al, USA, 1980 (37)	Prospective study, in-patients (no ICU) Descriptive PK	IV gentamycin 2/2.5 mg/kg/dose	According to kidney impairment level (see Paragraph “Aminoglycosides-Children” in text)	23 23	Kidney dysfunction (PD)y dysfunction	1	NR
Lanao et al, Spain, 1981 (38)	Prospective study, in-patients (no ICU) Descriptive PK	IV amikacin 7.5 mg/kg/dose	NR	10/18	Kidney dysfunction (CrCL 5–70 ml/min x1.73 m^2^)	1	NR
**Children and Adolescents**							
Yoshioka et al, Japan, 1978 (36)	Prospective study, in-patients (no ICU)	IM gentamycin 1 mg/kg/dose	NR	15	Kidney dysfunction	1	NR

Aminoglycosides are historically known to be nephrotoxic agents, and dosing adjustments for pediatric patients with renal impairment have been proposed since the late 1970's.

**Gentamicin** is used to treat systemic infections by Gram-negative bacteria, with optimal peak concentrations above 4 mg/L and desirable serum levels between 5 and 10 mg/L. Trough concentrations above 2 mg/L, together with a prolonged therapy duration, are at elevated risk of nephrotoxicity and ototoxicity (77–81). However, elevated peak levels seem not to correlate with a higher toxicity risk. Gentamicin has a dose-dependent post-antibiotic effect, which is enhanced by single-dose administration. Moreover, the therapeutic effect depends on a high C_Max_/MIC ratio. The molecule is chemically stable and excreted almost exclusively by glomerular filtration (in active form, 90%). It is eliminated through dialysis by at least 50%. Together with a narrow therapeutic window, these characteristics make dosing adjustment necessary in case of renal impairment (82).

Also **amikacin** is mainly eliminated through glomerular filtration, reaching high concentrations in urine. Renal impairment determines PK changes, affecting the elimination rate, with a need to adjust the dosing regimen to avoid antibiotic accumulation (83). Amikacin follows a two-compartment open-kinetic model. There is an increase in the elimination constant in children with a normal renal function, as it also happens for gentamicin. In children with an impaired renal function, there is a change in PK parameters which is especially manifest for a CrCl <30 ml/min/1.73 m^2^. This determines a lower rate of drug elimination, a delay in distribution, an increase in serum half-life and a risk of accumulation, if dosage intervals are not adjusted. The increase of the distribution volume appears to be more significant in children than adults.

##### Children

In the study by Sirinavin et al. on the use of IV gentamicin, data on 23 infants and children with various degrees of kidney failure indicated that gentamicin half-life could be estimated by multiplying the serum creatinine concentration by a factor of four (37). All participants received from 2 to 2.5 mg/kg as a first dose, with a dosing schedule over the first 24–48 h that followed adult guidelines and subsequent doses and administration intervals that followed PK calculations instead. The suggested schedule was one-half of the usual dose every half-life, three-quarters of the dose every two half-lives, or a full dose every three half-lives, and the choice of a regimen depends on the entity of renal impairment to avoid prolonged periods of sub-therapeutic concentrations. Administration schedules with dosing intervals longer than 24 h ensure a maximal bactericidal activity while reducing adverse effects. There was a statistically significant correlation between gentamicin half-life and serum creatinine concentration. (37).

Lanao et al. described the PK of amikacin in their prospective study on children with both normal and impaired renal function (38). Ten of the 18 included patients had various degrees of kidney impairment. All children received 7.5 mg/kg/dose in single bolus; then blood samples were withdrawn at 0, 0.5, 2.0, 4.0, 8.0, 12.0, and 16.0 h to determine blood levels. There was a linear correlation between the deterioration of renal function and a progressive decrease in the drug elimination rate. The study reported a decrease of the distribution constants, the overall elimination rate constant and serum clearance, with an increase of the distribution volume at steady state. The serum half-life, which was 1 h in children with normal kidney function, reached values of 14 h in children with severe renal impairment. The main modification in the PK of amikacin in children with kidney impairment was the decrease in the elimination rate, determining significant increases in the serum half-life. Thus, serum drug concentrations should be monitored systematically, together with renal function.

##### Children and Adolescents

Gentamicin administration at improper intervals may lead to either sub-therapeutic or potentially toxic serum concentrations. Yoshioka and colleagues reported the use of intramuscular gentamicin in 15 children and adolescents aged from 8 to 17, weighing 23 to 60 kg, with impaired kidney function. Peak levels and the change in blood levels up to the fourth dose were measured (36). A decline of serum concentrations appeared to be delayed in decreased kidney function, proportionally to the degree of renal damage. In particular, the half-life value increased sharply when CrCL values fell below 20 ml/min/1.73 m^2^. They also produced a diagram, plotting the half-life values against CrCL values, and calculated an equation of fitted regression line to predict the half-life for a known CrCL to adjust the dosing interval. Three patients were treated with the adjusted dosing schedule (administration intervals of 2.2, 3.5 and 3.7 times the estimated half-life, respectively). Peak blood levels, measured for repeated dose, remained within therapeutic levels, with no accumulation.

#### Cephalosporins

There were two retrieved studies on cephalosporins, cefepime and ceftolozane-tazobactam, respectively, both on children on HD, reporting PK data (Table 4).

**Table 4:** Cephalosporins.

**Author, country, year [reference]**	**Study design and setting**	**Anti-infective drug (s)** **Target** **Dosing regimen**	**Suggested dosing regimen**	**Number of patients (*n*)**	**Kidney dysfunction/AKI/ CKD +/- KRT (type)**	**Outcome(s)**	
						**PK parameters** **(yes 1, no 0)**	**clinical cure 0 therapy stop for toxicity 1 death 2 NR (*n*)**
**Children**							
Butragueño-Laiseca et al, Spain, 2020 (42)	Prospective study, in-patients (no ICU) PK model on Bayesian adjustment	IV ceftolozane-tazobactam, MDR *P. aeruginosa* pneumonia (+ ESBL *E. coli* bacteremia in Patient C) Patientt B: 36 mg/kg q8h;	NR	2/3	Patient B: Kidney dysfunction (eGFR of 22 mL/min/1.73 m^2^)	1 clearance 0.27 L/h, Vd_1_ = 1.13 L; Vd_2_ = 1.36;	0 (1/1)
		Patient C: 30 mg/kg q8h;			Patient C: Kidney dysfunction on CKRT	CKRT clearance 0.39 L/h, Vd_1_ = 0.74 L; Vd_2_= 1.17;	0 (1/1)
Stitt et al, USA, 2019 (41)	Retrospective study, (in-patients, PICU) Therapeutic drug monitoring	IV cefepime, empirical/*K. pneumonia*e pneumonia 48–64 mg/kg q6-12 h	NR	4	AKI CKRT (CVVHDF)	1	0 (4/4)

Cefepime is a fourth-generation cephalosporin active against Gram-negative bacteria, including *P. aeruginosa*. The pharmacodynamic parameter which makes bacterial killing optimal for cephalosporins is *f* T>_MIC_, which has to be 40% of the dosing interval for bacteriostasis and 70% for bactericidal activity, with increased bacterial killing for an *f* T>_MIC_ >100% of the dosing interval, according to *in vitro* studies (83, 84). *In vivo* studies on critically ill patients suggested as optimal free drug concentrations values ranging from one to four times the MIC (85–89).

Ceftolozane-tazobactam is a new antibiotic developed against the resistance mechanisms of *P. aeruginosa*, such as changes in porin permeability and upregulation of efflux pumps (90). *In vivo* studies proved favorable outcomes in more than 70% of patients with infections by *P. aeruginosa*. Ceftolozane is a small molecule with a low plasma protein binding rate (20%), and these characteristics suggest that it will be removed by CKRT (90). Approximately 95% is eliminated by glomerular filtration. Also, for ceftolozane, the PK/ pharmacodynamics parameter that best correlates with efficacy is the % *f* T>_MIC_.

##### Children

In the study by Butragueño-Laiseca and colleagues, data on dosing regimens of ceftolozane-tazobactam in three critically-ill children, of which two had kidney impairment, were reported (42). Patient B, of 19 months of age with hypoplastic left heart syndrome at Glenn stage, had pneumonia due to MDR *P. aeruginosa* and stage II KDIGO AKI with eGFR of 22 mL/min/1.73 m^2^; he received an adjusted dose of 36 mg/kg every 8 h. Patient C, of 9 months and on CKRT after heart transplantation, had pneumonia due to MDR *P. aeruginosa* and ESBL *E. coli* bacteremia, for which he received 30 mg/kg every 8 h. CKRT settings were blood flow 30 mL/min, substitution flow 160 mL/h, dialysis flow 250 mL/h, extraction 60 mL/h, and total effluent flow 470 mL/h. Drug PK was defined through patients' parameters by Bayesian estimation based on a population PK model. Dosing regimens were decided by the treating physicians, according to available data in the literature and each child's conditions. As a maximum exposure bound, the first PK criterion for dose selection was the 95th percentile of adult ceftolozane exposure (AUC_0−8_ = 628 (mg h/L) and C_max_ = 151 mg/L). Patient B exceeded it, with an AUC_0−8_ = 1,481 (mg h/L) and a C_max_ = 220 mg/L. As a potential efficacy bound, the second criterion was a minimum blood plasma concentration (C_min)_ = 16 for ceftolozane. This was met by all patients (C_min_ ≥ 16). Drug blood concentrations were measured and were always above the ceftolozane MIC. The terminal half-life of ceftolozane in patient B was double that of the patient without renal impairment because of a lower volume of distribution at steady state. For the hemodialyzed patient, the total estimated ceftolozane clearance rate during CVVHDF (0.39 L/h) was about half of that for the patient with a normal renal function. For both patients B and C, the predicted individual PK profiles were within the 90% predicted intervals computed from a model with the same individual characteristics.

The PK of this drug had previously been reported for healthy adults or adults with renal impairment (91–93). In this population, the dose requires adjustment for CrCL ≤ 50 ml/min. A 2.5-fold increase in the AUC for ceftolozane was found in subjects with moderate kidney impairment, while for subjects with severe kidney impairment, the AUC for ceftolozane increased by 4.4-fold. Data on PK for children and adolescents are limited (94–98), finding that exposures estimated through a non-compartmental analysis were similar to those reported for adults but with no data regarding children with renal impairment or on dialysis. Results for the patient with AKI suggested that patients with moderate to severe renal impairment should require a decrease in the dose or a lower frequency of administration. As regards CKRT, an *ex-vivo* experiment did not report significant differences in the sieving coefficient between hemofilter types, ultrafiltrate flow rates or KRT modality (97). Increases in effluent flow and dialysis flow directly increase trans-membrane clearance, especially in children using moderate and high effluent flows (99). The extraction ratios and sieving coefficients found in this study were similar to previous adult findings. Moreover, the total estimated ceftolozane clearance rate during CVVHDF was half of the child with a normal renal function. However, dosing recommendations for CKRT do not apply to intermittent hemodialysis for PK differences. All patients reached a high AUC at steady-state and a % *f* T>_MIC_ of 30% of an 8-h dosing interval for a MIC ≤ 4 mg/L. A dose of 10 mg/kg every 8 h may be sufficient in patients with AKI, with a dose reduction in line with the one proposed for adults with AKI, while a dose of 30 mg/kg every 8 h can be considered for children on CKRT and a high effluent rate.

The retrospective study by Stitt et al. reported PK data on four critically ill children (three male and one female, aged 0.5–5 years) admitted to PICU, on continuous venovenous hemodiafiltration (CVVHDF) for AKI, fluid overload and to aid in ammonia clearance, treated with intravenous cefepime, empirically (three cases) or targeting *K. pneumoniae* pneumonia (one case) (41). Cefepime doses ranged from 48 to 64 mg/kg every 6–12 h. Drug peak, mid-interval and trough concentrations were measured at steady state. All children had a low or absent urine output during therapeutic drug monitoring, thus minimizing the effect of intrinsic renal clearance on cefepime PK. The minimum starting clearance dose of KRT was 2,000 ml/1.73 m^2^/h with 50% diffusive and 50% convective clearance. Optimal free trough concentrations were reached in three patients (100% *f* T>1_XMIC_, with desirable free trough concentrations ranging from one to four times the MIC for cefepime), with one of them, with the lowest CVVDHF clearance and blood flow rates, reaching 100% *f* T>4_XMIC_. One patient achieved 98.3%100% *f* T>1_XMIC_. There were no reported adverse effects directly linked to the drug. Their retrospective study provides the first data on cefepime PK in children on CKRT, suggesting that the standard doses of 50 mg/kg every 12 h may not allow reaching easily aggressive pharmacodynamics targets of 100% *f* T>_1−4XMIC_ in this special population. Despite the limit of only four included patients, their data suggest the wide use of therapeutic drug monitoring as a valuable tool to reach more aggressive pharmacodynamics targets, especially in case of higher prescribed clearance. The calculated volume of distribution and drug half-life in these patients were similar to values for patients without renal impairment, but it is not possible to assume any more definitive link between delivered CVVHDF clearance and cefepime clearance due to data being limited.

#### Lipopeptides

Both retrieved studies on lipopeptides were case reports on daptomycin, with data on children on PD with descriptive PK and HD, respectively (Table 5).

**Table 5:** Lipopeptides.

**Author, country, year [reference]**	**Study design and setting**	**Anti-infective drug (s)** **Target** **Dosing regimen**	**Suggested dosing regimen**	**Number of patients (n)**	**Kidney dysfunction/AKI/ CKD +/- KRT (type)**	**Outcome (s)**	
						**PK parameters** **(yes 1, no 0)**	**clinical cure 0 therapy stop for toxicity 1 death 2 NR (n)**
**Children**							
Morris et al. UK, 2017 (44)	Case report, in-patient (PICU) Descriptive PK	IV daptomycin, *S. epidermidis* bacteremia 8 mg/kg every 48 h	NR	1	AKI (PD)	1	0 (1/1)
Chan et al, USA, 2012 (43)	Case report, in-patient (no PICU)	IV daptomycin, *VRE E. faecium* peritonitis and bacteremia; initial dose: 6 mg/kg every 48; empirically increased to 8 mg/kg every 48 h for 113 days;	NR NR	1 1	Kidney failure (HD) (PD)y failure (HD)	0	0 (1/1)

Daptomycin is a concentration-dependent antibiotic, primarily excreted unchanged by the kidney. Its efficacy is related to AUC/MIC and maximum plasma concentration-to-MIC ratios. It is generally well-tolerated, and monitoring of serum CPK concentrations is required for reported cases of myositis, rhabdomyolysis and myoglobinemia. Measurement of serum CPK is mandatory for patients with any degree of kidney impairment. To date, there are no recommended doses for children with kidney failure. Dosing recommendations for impaired kidney function follow adult patients (extension of the dosing interval to every 48 h for estimate CrCL of <30 mL/min, with or without dialysis, supported by population pharmacokinetic modeling). Clearance, in general, is expected to be higher in young children.

##### Children

Morris et al. reported the case of an 8-year-old 17-kg girl with congenital heart disease multiorgan failure with AKI, requiring continuous peritoneal dialysis, that presented *S. epidermidis* bacteremia, successfully treated with IV daptomycin (44). The chosen dose was 8 mg/kg/dose every 48 h (for concerns for resistance) for 19 days in total, and PD scheme had 10 ml/kg cycle volumes and hourly cycles. The MIC for daptomycin was 0.125 mg/L. There was a good, rapid clinical and microbiological response, with a good safety profile. Drug concentrations were measured using a validated high-performance liquid chromatography assay. Peak and trough serum concentrations were 68 mg/L and 14.6 mg/L, respectively (target trough <20 mg/L). They demonstrated in their study that an IV dosage of 8 mg/kg every 48 h during continuous PD achieved peak concentrations similar to those achieved at steady state in adults with CrCL of more than 30 mL/min treated with 6 mg/kg every 24 h. Moreover, exposure on the second day after dosage was in line with the IV suggested dose of 6 mg/kg every 48 h for adults on continuous peritoneal dialysis. There is a correlation between toxicity and trough levels, with a chance that CPK elevation will be significantly higher with a trough serum concentration ≥ 24.3 mg/L. According to the elimination half-life, the authors suggested that serum trough concentration should be performed before the third dose in pediatric patients on continuous PD when steady state is likely to have been achieved with doses every 48 h, with a recommended trough concentration of <20 mg/L. Moreover, they recommended serum peak concentration measurement and AUC/MIC calculation only in case of concern about clinical efficacy or reduced probability of pharmacodynamic target attainment for MICs higher than 0.5 mg/L (44).

Chan et al. described a case of peritonitis and bacteremia in a 5-year-old girl that underwent a liver transplant and developed renal failure, requiring hemodialysis (43). She was first empirically treated with vancomycin and piperacillin-tazobactam (doses not specified but adapted for kidney function). Vancomycin-resistant *E. faecium* (VRE) was found in peritoneal fluid culture, and the therapy was first shifted to meropenem and linezolid (dose not specified), with subsequent resolution of fever. A second culture was found positive for linezolid-resistant VRE, so current therapy was discontinued and daptomycin was initiated and continued for 113 days at a dose of 6 mg/kg every 48 h, then empirically increased to 8 mg/kg for dialytic treatment and persisting positivity of cultures, for concern for reduced potency against *E. faecium* as compared to *S. aureus*. Drug concentrations were measured before HD after dose increase. After 45 min since the end of infusion, the serum concentration would approximate the MIC at steady state of adult patients with *S. aureus* bacteremia and a normal renal function receiving 6 mg/kg. The drug was well tolerated with normal creatine phosphokinase (CPK) levels. Clinical cure was achieved, and the patient eventually underwent combined liver and kidney transplant.

#### Penicillins

One case report on therapeutic drug monitoring of temocillin in a preterm newborn was retrieved (Table 6).

**Table 6:** Penicillins.

**Author, country, year [reference]**	**Study design and setting**	**Anti-infective drug (s)** **Target** **Dosing regimen**	**Suggested dosing regimen**	**Number of patients (n)**	**Kidney dysfunction/AKI/ CKD +/- KRT (type)**	**Outcome (s)**	
						**PK parameters** **(yes 1, no 0)**	**clinical cure 0 therapy stop for toxicity 1 death 2 NR (n)**
**Neonates**							
Dumangin et al, France, 2020 (47)	Case report, in-patient (no ICU) Therapeutic drug monitoring	IV temocillin, pyelonephritis by ESBL *E. cloacae* continuous infusion, loading dose: 3.5 mg/kg, followed by maintenance dose: 10 mg/kg/day	dosing a djustment with reduction by 0.6-, 0.3-and 0.1-fold for a CrCL of 60, 30 and 10 mL/min/1.73 m^2^	1	Kidney dysfunction eGFR 11 to 17 mL/min/1.73m^2^, CrCl 14 mL/min/m^2^	1	0 (1/1)

Temocillin is an old carboxypenicillin, mainly used for complicated urinary tract infections by susceptible ESBL-producing Enterobacteriaceae, currently available in France, Belgium, Luxembourg, the UK and Iran for urinary and low respiratory tract infections and bacteremia. It imposes no selection pressure on gram-positive organisms, anaerobes and *P. aeruginosa* (100). Over the last decade, a renewed interest in temocillin has emerged for its potential use as a carbapenem-sparing agent (101). However, its spectrum appears to be limited to *Enterobacteriaceae*, with stable MICs over the past few years (2–32 mg/L, cut-off of 16 mg/L) (99). EUCAST set clinical breakpoints of ≤ 0.001 mg/L for susceptible and > 16 mg/L for resistant *E. coli, Klebsiella spp*. and *Proteus mirabilis*. It is mainly eliminated in the urines in the unchanged form, for 80% of a dose, with delay in renal impairment. The recommended dose for the adult and pediatric population with normal renal function is respectively 4–6 g/day and 25–50 mg/kg/day. In particular, the reported IV dose for children without kidney impairment for acute pyelonephritis is 25 mg/kg twice a day. In adults with impaired kidney function, there is a significant positive linear relationship between drug clearance and CrCL and a 3.7-fold increase of AUC value in individuals with severe kidney dysfunction. Also, an increase of half-lives from 5 to 30 h was reported in patients with kidney impairment (102, 103).

##### Neonates

Dumangin et al. described the safe and effective use of temocillin after dosage adjustment in a 7-month-old ex-preterm infant with ESRD to treat a urinary tract infection (UTI) due to a susceptible ESBL-producing *Enterobacter cloacae* (MIC = 6 mg/L). Temocillin was administered in this patient with a loading dose of 3.5 mg/kg, followed by a maintenance dose of 10 mg/kg/day through continuous infusion. The loading dose was empirical and corresponded to one-third of the maintenance dose. The latter was 5-fold lower than the usual pediatric recommended dose, as described in the summary of product characteristics for infections by ESBL-producing *Enterobacteriaceae*. Treatment duration was 10 days, with clinical cure. Urinary and plasmatic concentrations of drugs were monitored. The estimated urinary concentration at the end of therapy was 38.7 mg/L. 100% *f* T_>MIC_ was achieved in urine since temocillin elimination in urine is predominant (52% to 92%) and delayed in kidney dysfunction. The accumulation rate in urine was similar to adults with severe kidney insufficiency. Plasma levels were 47.0 and 61.8 mg/L after 6 and 10 days of treatment, respectively, with an estimated free concentration of 7.0 and 9.2 mg/L. Free plasma concentrations were estimated to be above the MIC during the last 4 days of treatment, with a minimum of 40% *f* T_>MIC_ (47). Temocillin dosing was adjusted with a reduction by 0.6-, 0.3-and 0.1-fold for a CrCL of 60, 30 and 10 mL/min/1.73 m^2^, respectively, according to the available data from the literature, as reported above. Other authors proposed increasing the dosing interval maintaining the same dose as in normal patients (103). Larger clinical and PK studies on temocillin are required in order to determine the optimal regimens for infants and children with kidney impairment.

#### Polymixins

The only retrieved study was a case report also providing PK parameters on colistin use (Table 7).

**Table 7:** Polymixins.

**Author, country, year [reference]**	**Study design and setting**	**Anti-infective drug (s)** **Target** **Dosing regimen**	**Suggested dosing regimen**	**Number of patients (*n*)**	**Kidney dysfunction/AKI/ CKD +/- KRT (type)**	**Outcome (s)**	
						**PK parameters** **(yes 1, no 0)**	**clinical cure 0 therapy stop for toxicity 1 death 2 NR (*n*)**
**Adolescents**							
Healy et al, USA, 2011 (48)	Case report, in-patient (no ICU) Descriptive PK	IV colistin, MDR *A. baumannii* burn wound sepsis 2.5 mg/kg every 24 h;	NR	1	AKI CKRT (CVVHD)	1 total body CL 5.2 ± 1.7 ml/min	2 (1/1)

A renewed interest in **colistin** (polymixin E) has been recently determined by the threat of emerging MDR Gram-negative bacteria. IV colistin as sulfate salt has been available since the 1950's, but with historically limited use, due to potential nephrotoxicity. The IV formulation was eventually changed to colistimethate sodium, an inactive pro-drug, which is presently used and less toxic.

##### Adolescents

Healy and colleagues provided valuable colistin PK information in their 2011 report on an adolescent with extensive burn injury by MDR *A. baumannii* with septic shock and on CVVHDF, receiving a standard dose for moderate renal impairment (2.5 mg/kg/day). C_max_, Cmin, AUC0–24 h, total body clearance, and elimination half-lives (t _1/2_) of colistin were 3.6 ±1.0 mg/L, 0.9 ± 0.5 mg/L, 47.1 ±14.4 mg/hr/L, 5.2 ±1.7 ml/min, and 12.3 ±9.4 h (mean ±SD), respectively (48).The elimination half-life of colistin during CVVHD was prolonged (13.4 ±5.1 h) while corresponding values on dialysis were 9.4 ±4.6 h. Serum concentrations were at or above the MIC for >90% of treatment. The average peak to MIC ratio was adequate, with a dose of 2.5 mg/kg every 24 h. Despite adequate serum levels and repeat negative cultures, the patient eventually died after developing acidosis, coagulopathy and vasopressor-dependence (48).

Recent evidence from the adult population suggested both peak/MIC and (AUC)/MIC ratios as important parameters for bacterial eradication (104–106). Therefore, dosing should be adjusted to the organism MIC, even though the product literature does not provide such recommendations. Moreover, to avoid underdosing in the early phases of treatment, dosing should follow the actual body weight when adequate drug concentrations are critical. In the report, doses according to an ideal patient weight would have led to underdosing without achievement of adequate peak/MIC and (AUC)/MIC ratios. The principal means of excretions of colistin is thought to be extra-renal, with significantly reduced clearance in critically ill patients (107, 108). Data from this study are in line with the literature on adults also in reporting some degree of drug removal by dialysis (107). The kidney function appears not to alter the individual dose size, but it is advisable, according to these preliminary data, to use it to guide the frequency of administration, to avoid accumulation and nephrotoxicity risk. Therefore, the suggested minimum dose to achieve adequate peak/MIC and (AUC)/MIC ratios for organisms with low MICs is 2.5 mg/kg, while a dose of 5 mg/kg may be more appropriate for higher MICs (1–2 mg/L). In case of decreased kidney function, the administration intervals should be lengthened, but not longer than once every 24 h. Studies on colistin use in younger patients or even neonates are lacking and therefore needed.

#### Combined Antibiotic Therapy

The two retrieved studies were an RCT also reporting PK data on children and adolescents and a case report on an adolescent. Both dealt with the intraperitoneal treatment of CPD-related peritonitis (Table 8).

**Table 8:** Combined antibiotic therapy.

**Author, country, year [reference]**	**Study design and setting**	**Anti-infective drug(s)** **Target** **Dosing regimen**	**Suggested dosing regimen**	**Number of patients (*n*)**	**Kidney dysfunction/AKI/ CKD +/- KRT (type)**	**Outcome(s)**	
						**PK parameters** **(yes 1, no 0)**	**clinical cure 0 therapy stop for toxicity 1 death 2 NR (*n*)**
**Children**							
Schaefer et al, Germany, 1999 (39)	RCT, in/out-patients (no ICU) Descriptive PK	IP glycopeptide (vancomycin or teicoplanin)/ceftazidime, peritonitis intermittent vs. continuous IP infusion; (Ia) continuous infusion vancomycin (30 mg/L)/ceftazidime (125 mg/L); (IIa) continuous infusion teicoplanin (20 mg/L)/ceftazidime (125 mg/L); (Ib) intermittent infusion vancomycin (second loading dose 30 mg/kg)/250 mg/L; (IIb) intermittent infusion teicoplanin (second loading dose 15 mg/kg)/250 mg/L	NR	90 (168 episodes of peritonitis, 27 relapses, total 195 episodes)	CKRT (PD)	1	Overall: 0 (141/168) (168 episodes of peritonitis, 27 relapses) (Ia) 0: (22/25 Gram+); (7/7 Gram-); (6/8 sterile or mixed culture) (IIa) 0: (31/31 Gram+); (5/7 Gram-); (7/7 sterile or mixed culture)
							(Ib) 0: (22/22 Gram+); (8/10 Gram-); (8/8 sterile or mixed culture) (IIb) 0: (29/31 Gram+); (0/1 Gram-); (8/9 sterile or mixed culture)
**Adolescents**							
Shetty et al. USA, 2005 (40)	Case report, in-patient (no PICU)	IP cefazolin/gentamicin, empirical cefazolin (500 mg/l) and gentamicin (8 mg/l) IP as “loading” dose and 125 mg/l and 4 mg/l, respectively, for maintenance;	NR	1	Kidney failure (PD)	0	0 (1/1)
		IP ceftriaxone, *N. mucosa* PD-associated peritonitis (125 mg/l dialysate) for 2 weeks;	NR				0 (1/1)

Historically, primary treatment response rates in adults and children with PD-related peritonitis ranged between 60 and 85% (109) for insensitivity to the first-line therapy, persistent organisms in the catheter biofilm, and a lack of standardized guidelines for diagnosis and therapy (110). A 1993 consensus provided recommendations for management based on a meta-analysis of the literature, proposing the combination of intraperitoneal vancomycin and ceftazidime or vancomycin and an aminoglycoside as empirical therapy (111). The suggested vancomycin regimen of two boluses seven days apart considered its prolonged half-life in CKD. An intermittent administration of ceftazidime was also proposed for its diminished elimination in uremia. However, this approach was not readily accepted in clinical practice, also for the lack of controlled studies.

##### Children

Schaefer et al. led a large RCT for evaluation of efficacy, safety and acceptance of combined intraperitoneal treatment with vancomycin/teicoplanin and ceftazidime, administered either intermittently or continuously in 90 pediatric patients with continuous peritoneal dialysis (CPD)-related peritonitis (195 episodes in total), proving the equivalence of continuous and intermittent treatment (39). Children randomized to continuous treatment received an intraperitoneal loading dose of glycopeptide and ceftazidime (Table 8), followed by maintenance doses added to each dialysate bag. In the intermittent administration groups, the glycopeptide was administered in two loading doses seven days apart and ceftazidime during one dialysis cycle per day (Table 8). Treatment response was evaluated after 60 h and after seven days of treatment by changing Disease Severity Score (DSS) and clinical decision. In case of satisfactory response at the initial (60 h) treatment, ceftazidime was discontinued in a gram-positive infection and the glycopeptides in case of a gram-negative infection. If clinical improvement was considered insufficient, the antibiotic therapy was tailored individually. Twenty-seven relapses were reported within 4 weeks after the end of therapy. Clinical cure rates by treatment group are reported in Table 8. In Gram-positive infections, there were no differences between continuous (RR 1.17; 95% CI, 0.51–2.67) and intermittent administration (RR 0.83; 95% CI, 0.30–2.27) and between vancomycin (RR 1.41; 95% CI, 0.58–3.45) and teicoplanin (RR 0.70; 95% CI, 0.28–1.76) as regards the relative risk for primary treatment failure according to DSS. Unresponsive cases to initial treatment were due to *S. aureus*. In Gram-negative peritonitis, germs with *in vitro* resistance to ceftazidime were cultured in nearly all cases of lack of response by DSS.

Eradication of the causative germ from the dialysate was observed significantly more often in the continuous than in the intermittent treatment groups both after 60 h (94 vs. 67%, *P* < 0.001) and after seven days (99 vs. 90%, *P* = 0.03). In four patients from the continuous administration group, elevated plasmatic levels of glycopeptides were found, while in two intermittently treated children underdosing was observed (but with adequate clinical treatment and not increased relapse rates). Plasmatic levels of intermittently administered vancomycin were inversely correlated with residual GFR but with no relation between blood levels and GRF for intermittent teicoplanin. There was a significant decrease in residual GFR during peritonitis (*p* = 0.005), showing no differences in relation to the type of glycopeptide or administration. The Authors demonstrated the validity of intermittent glycopeptide administration in children with ESRD, as all patients showed acceptable and safe blood levels during the first 60 h of treatment. Moreover, there were no secondary treatment failures due to inadequate tissue levels.

The nephrotoxic action of glycopeptides remains a concern even in the case of dialysis because of residual kidney function contributing to total solute and water clearance. In this study, residual kidney function deteriorated, but it was not possible to recognize glycopeptide therapy or peritonitis itself as the leading cause. Teicoplanin levels, because of a higher plasma protein binding, were not affected by residual kidney function on the third day of treatment and were more stable after many days after the administration on the loading dose, unlike vancomycin levels. The routine use of glycopeptides for the treatment of CPD-related infections should be in any way discouraged for the increasing prevalence of VRE after their widespread use. As for ceftazidime in combination therapy, it was used for its broad coverage against Gram-negative organisms, including *Pseudomonas*, avoiding the toxicity of aminoglycosides. It was also chosen for its outstanding bidirectional transperitoneal diffusion and prolonged half-life in ESKD, so once-daily intraperitoneal administration appears an appropriate administration regimen. In this study, it was administered during one prolonged dwell period or over continuous administration. The treatment response was judged discrepantly by clinical decision and DSS, but the delayed bacterial elimination in patients receiving intermittent treatment did not affect the final treatment outcome or the risk of relapse.

##### Adolescents

The other retrieved study was a case report by Shetty et al. on the use of empirical intraperitoneal combined therapy to treat PD-related peritonitis by *N. mucosa* in a 17-year-old male adolescent with ESKD (40). There are few reported cases in adults with ESKD, and there is no specific guideline for the optimal treatment in children with kidney dysfunction. The patient was first treated empirically with intraperitoneal **cefazolin** (500 mg/L) and **gentamicin** (8 mg/L) as a loading dose, followed by a maintenance dose of 125 mg/L and 4 mg/L, respectively. Prompt clinical response was evident after 48 h. Before discharge, after 6 days of therapy, he was shifted to intraperitoneal ceftriaxone (125 mg/L) for 2 weeks as targeted therapy. Clinical and biochemical cures were confirmed at one-month evaluation.

#### Antivirals

We retrieved three articles on antiviral use in children with renal impairment, of which two case reports (on a child and a newborn, respectively, both reporting PK data) and one prospective study on children, all on CKRT. They described the safe and effective use of three different molecules in intensive care unit contexts for critical pediatric patients with kidney dysfunction, including neonates (Table 9).

**Table 9:** Antivirals.

**Author, country, year [reference]**	**Study design and setting**	**Anti-infective drug (s)** **Target** **Dosing regimen**	**Suggested dosing regimen**	**Number of patients (*n*)**	**Kidney dysfunction/AKI/ CKD +/- KRT (type)**	**Outcome (s)**	
						**PK parameters** **(yes 1, no 0)**	**clinical cure 0 therapy stop for toxicity 1 death 2 NR (*n*)**
**Neonates**							
Cies et al, USA, 2015 (34)	Case report, in-patient (ICU) Therapeutic drug monitoring	IV/in dialysate acyclovir, disseminated HSV-1 infection 30 mg/kg/dose every 8 hours, then continuous infusion 5.5 mg/L added to dialysate solution.	NR	1	Kidney failure (CKRT)	1	0 (1/1)
**Children**							
Shetty et al, USA, 2011 (33)	Case report, in-patient (ICU) Therapeutic drug monitoring	IV peramivir, H1N1 influenza pneumonia 2.2 mg/kg/day, then 5.4 mg/kg/day For two weeks;	NR	1	Kidney failure (CRKT)	1	0 (1/1)
Ozsurekci et al, Turkey, 2021 (35)	Prospective study, in-patients (PICU)	Oral favipiravir, MIS-C in Covid-19 infection	22–35 kg: 1,200 mg (initial and 8 h dose), then 600 mg (16 h dose) dose; on day 2 and 4: 600 mg every 12 h 46–55 kg: 2,000 mg (initial and 8 h dose), then 1,000 mg (16 h dose); on day 2 and 4: 1,000 mg every 12 h >55 kg: 2,400 mg (initial and 8 h dose), then 1,200 mg (16 h dose); on day 2 and 4: 1,200 mg every 12 h	2/11	Kidney dysfunction (eGFR <30 ml/min/1.73 m^2^) (CKRT)	0	0 (2/2)

##### Neonates

Acyclovir is the first choice treatment for severe HSV infections in newborns. The drug is mainly eliminated through the kidney with a combination of glomerular filtration and tubular secretion (112). The molecule is small, water-soluble, with a low protein binding, making it readily eliminated by all kinds of dialysis. Acyclovir has a time-dependent killing, correlating efficacy and time above the MIC of serum concentration. For this reason, a continuous infusion is a valuable option. Concentrations in the CSF correspond to 30–50% of plasma concentrations. Dosing should aim at maintaining plasma levels of 3 mg/L or above to ensure CSF concentrations of at least 1 mg/L for optimal outcomes, below 50–70 mg/L, to avoid neurotoxicity (112). Continuous infusion administration is a safe and effective option in case of failure of intermittent administration (113). Nevertheless, there are no data in the literature regarding the use and PK of acyclovir during CKRT. Standard dosing recommendations are at risk of therapeutic failure.

Cies et al. reported data on the use of **acyclovir** to treat disseminated HSV-1 infection in a 14-day-old neonate with fulminant kidney failure requiring CKRT (34). Acyclovir was already administered intravenously at the standard dose of 20 mg/kg/dose every 8 h, but after conversion to ECLS and CKRT, the dose was increased to 30 mg/kg/dose every 8 h. Because of persistently high viral loads, the continuous infusion was started, with acyclovir being added to the dialysate solution for CKRT (5.5 mg/L), in order to maintain plasma concentrations of at least 3 mg/L and thus at least 1 mg/L in the cerebrospinal fluid (CSF). Serum concentrations at 24 and 72 h after continuous infusion start were adequate for this purpose.

In this article, the existing literature on acyclovir use for adults on CKRT was used as a model. The practice of adding anti-infective medicines to the dialysate solution is not new, usually common in peritoneal dialysis, but not as well-represented in hemodialysis. Modern dialysis filters, circuits and machines may make standard adult dosing recommendations not appropriate to date (34). The Authors chose the intra-dialytic administration route to minimize fluctuations from dialysis clearance based on flow rates. This strategy was successful and confirmed by serum concentrations measurements at 24 and 72 h. Drug PK may be affected by sequestration in the circuit, increased volume of distribution and increased clearance during CKRT, with all available data being derived from adult studies. *Ex-vivo* models/circuits may indeed be helpful for the improvement of dosing regimens and CKRT patient outcomes.

##### Children

Peramivir has been experimentally considered an alternative for oseltamivir-resistant strains, but there are no further data on the use of this drug in children.

Shetty et al. reported the case of a 10-year-old male renal transplant recipient that developed renal failure requiring ECMO and CKRT for oseltamivir-resistant 2009 H1N1 pneumonia (33). He was successfully treated with IV peramivir for 2 weeks at an initial dose of 2.2 mg/kg/day. Over the first 72 h of therapy, peak and trough drug levels were assessed, with a subsequent dose increase to 5.4 mg/kg/day to reach a target trough concentration of >1,500 ng/mL. There were no reported adverse effects, with clinical cure and kidney failure improvement by day 13 of treatment. It was administered through an Emergency New Drug Application; the dosage was adapted from adult dose and adjusted in order to achieve adequate target trough concentrations, according to peak and trough levels at 72 h. In addition, the patient's recovery and viral clearance may have been helped because immunosuppressive therapy for renal transplant was discontinued when CKRT was started to sustain kidney function, even facing the risk of graft rejection (33).

Favipiravir is a nucleotide analog inhibiting RNA polymerase approved against influenza in China and Japan. The molecule is hydrophilic, with a protein-bound fraction of 50% and a distribution volume of 15–20 L. It is administered orally for its excellent bioavailability (100%) and is quickly absorbed with a t_max_ from 30 to 60 min (114). It is mainly eliminated through the kidney, and it has a time- and dose-dependent PK. Serum concentrations may significantly be affected by an impaired kidney function, but there is a lack of studies on drug metabolism in the literature, and there are no existing recommendations for its safe use in children with kidney dysfunction.

Favipiravir was used with no dose adjustment and no measurement of plasma levels by Ozsurekci and colleagues in two out of 11 critically ill children diagnosed with the multisystem inflammatory syndrome in children (MIS-C) after Covid-19 infection, on CKRT, with eGFR <30 ml/min/1.73 m^2^ (35). Doses followed weight as per the recommendations for Ebola (Table 9), without any dose adjustment for kidney dysfunction. Favipiravir use was safe and effective in this category of patients. Other studies in adults recommended dose adjustment in case of kidney impairment (115) due to the accumulation in reduced eGFR of an inactive metabolite, which might be related to toxicity. However, there is a lack of data on the optimal dose and adjusted dose to eGFR for both adults and children (116). In the latter especially, the drug is used off-label. As reported by Ozsurekci, there are no suggestions from the literature about skipping the loading dose in patients with GFRs from 30 to 50 m/min/1.73 m^2^, as the expected increase of two- to three-fold in plasma concentrations is considered safe (117). Larger studies are needed to clarify this aspect.

#### Antifungals

There were three retrieved studies on the use of antifungal agents: a prospective study on amphotericin B different preparations and a case report on the use of combined antifungal agents, both on neonates with kidney dysfunction, and a case report with descriptive PK on the use of fluconazole in an adolescent on CVVH (Table 10).

**Table 10:** Antifungals.

**Author, country, year [reference]**	**Study design and setting**	**Anti-infective drug (s)** **Target** **Dosing regimen**	**Suggested dosing regimen**	**Number of patients (*n*)**	**Kidney dysfunction/AKI/ CKD +/- KRT (type)**	**Outcome (s)**	
						**PK parameters** **(yes 1, no 0)**	**clinical cure 0 therapy stop for toxicity 1 death 2 NR (*n*)**
***Neonates***							
Linder et al, Israel, 2003 (45)	Prospective study, in-patients (NICU)	IV amphotericin B, *Candida* bloodstream infections for CrCL <1.2 mg/dL 1 mg/kg/day	NR	34/56	Kidney dysfunction	0	0 (29/34; 67.6% in monotherapy; 100% in addition to a second antifungal agent); 2 (5/34)
		IV liposomal amphotericin, *Candida* bloodstream infections for CrCL ≥ 1.2 mg/dL 5 mg/kg/day	NR	6/56			0 (5/6, 83.3% in monotherapy; 83.3% in addition to a second antifungal agent); 2 (1/6)
		IV amphotericin B colloidal dispersion, *Candida* bloodstream infections for CrCL ≥ 1.2 mg/dL 3 mg/kg/day on first day, then 5 mg/kg/day;	NR	16/56			0 (14/16, 57.1% in monotherapy; 92.8% in addition to a second antifungal agent); 2 (2/16)
Cheng et al, Taiwan 2010 (46)	Case Report, in-patient (no-ICU)	IV amphotericin B lipid formulation, *Candida parapsilosis* peritonitis 4 mg/kg/day	NR	1	Kidney failure (PD, then CVVHF)	0	0 (1/1)
		IP fluconazole, *Candida parapsilosis* peritonitis 0.1 mg/mL	NR				0 (1/1)
		Oral 5-flucytosine, *Candida parapsilosis* peritonitis 50 mg/kg/day	NR				0 (1/1)
		IV caspofungin, *Candida parapsilosis* peritonitis 25 mg/m^2^/day, then 40 mg/m^2^/day	NR				0 (1/1)
		IV fluconazole, *Candida parapsilosis* peritonitis 10 mg/kg/day	NR				0 (1/1)
**Adolescents**							
Oualha et al, France, 2019(49)	Case Report, in-patient (PICU) Individual PK model development	IV fluconazole, biliary and peritoneal candidiasis (*C. parapsilosis*) 10 mg/kg per day (600 mg once daily for 63 days)	loading dose of 900 mg; maintenance dose of 600 mg twice daily	1	Kidney failure (CVVHD)	1 Estimated CL 2.79 L/h; estimated V 30 L; estimated dialysate CL 1.64 L/h (52% of the total CL of fluconazole).	2 (1/1)

*AKI, acute kidney injury; CKD, chronic kidney disease; KRT, kidney replacement therapy; PK, pharmacokinetics; AUC, area under the curve; CL:clearance; MIC, minimum inhibitory concentration; Vd, distribution volume; t_1/2_, elimination half-life; C_max_, peak serum concentration; C_min_, minimum serum concentration; NR, not reported; PICU, Pediatric Intensive Care Unit; IV, intravenous; y:years; CKRT, continuous kidney replacement therapy; NICU, Neonatal Intensive Care Unit; IP, intraperitoneal; PD, peritoneal dialysis; IQR, interquartile range; h, hours; MRSA, Methicillin-resistant Staphylococcus Aureus; ICU, Intensive Care Unit; CrCL, creatinine clearance; HD, hemodialysis; CVVHD, continuous veno-venous hemodalysis; CVVHDF, continuous veno-venous hemodiafiltration, ALL, acute linfoblastic leukemia; HSV, herpes simplex virus; MIS-C, multiinflammatory syndrome in children; eGFR, estimated glomerular filtration rate; IM, intramuscular; ESBL, extended spectrum beta lactamase; MDR, multidrug resistant*.

Amphotericin B is widely used to treat *Candida* bloodstream infections but with potential nephrotoxicity. Amphotericin B colloidal dispersion and liposomal amphotericin B have been used in adults with impaired kidney function or concomitant use of nephrotoxic agents.

Fluconazole is a safe and effective triazole antifungal agent with concentration-dependent activity. Reported optimal pharmacodynamic end-points are a free-drug AUC curve from 0 to 24 h (fAUC0–24/MIC) ratio >100, a C_peak_ range from 16 to 32 mg/L, and a C_trough_ >10 mg/L have been suggested as the appropriate pharmacodynamic end points. It has a low protein binding and molecular weight, determining diffusion through the dialysis membrane (49, 118–120).

##### Neonates

Linder et al. enrolled 56 NICU patients (of which 36 ELBW neonates) with *Candida* bloodstream infection in a prospective study comparing the effectiveness and tolerability of three different amphotericin B preparations (45). Thirty-four neonates had serum CrCL of < 1.2 mg/dl and received **amphotericin B** at a dose of 1 mg/kg/day; patients with serum CrCl ≥ 1.2 mg/dl received either **liposomal amphotericin B** at a dose of 5 mg/kg/day (6 patients) or **amphotericin B colloidal dispersion** at a dose of 3 mg/kg/day on the first day, then 5 mg/kg/day (16 patients). There were no significant differences in mortality and no deterioration of kidney function. In case of positive urine cultures or fungus ball, a second antifungal agent was added directly, or introduced for persistent infection for more than 10 days, clinical signs and/or persistent thrombocytopenia. After 14 days from the last positive blood culture, therapy was discontinued in uncomplicated cases. Sterilization of the blood was reported in 67.6% of patients treated with amphotericin B, in 83.3% of patients treated with liposomal amphotericin B in 57.1% of those treated with amphotericin B colloidal dispersion when used as monotherapy. After adding a second antifungal agent, success rates were 100%, 83.3%, and 92.8% in the three groups. Liposomal amphotericin B and amphotericin B colloidal dispersion were safe and effective in premature infants with kidney dysfunction, as effective as conventional amphotericin B for eradicating invasive *Candida* infections. Therefore, the lipid formulation of amphotericin B is among the drugs of choice for invasive *Candida* infections in infants with kidney failure.

Cheng et al. reported the successful use of a combination of antifungal agents to treat *C. parapsilosis* peritonitis in a full-term neonate, requiring PD for kidney failure after *E. coli* sepsis, treated with broad-spectrum antibiotics. IV amphotericin B lipid formulation was started at a dose of 4 mg/kg/day, together with intraperitoneal fluconazole at 0.1 mg/mL to enhance peritoneal penetration, but without success. (46). The dialysis tube was removed after isolation of *C. parapsilosis* from blood and peritoneal dialysate; CVVHF was started. Oral 5-flucytosine (50 mg/kg/day) that is usually administered with amphotericin B or fluconazole for fungal peritonitis was also added, and its dose was adjusted to glomerular filtration rate (10–50 mL/min/1.73 m^2^), increasing the interval of administration from 12 to 24 h. Serum levels were maintained under 100 mg/L (118). IV caspofungin (25 mg/m^2^/day, then increased to 40 mg/m^2^/day) was started for concerns about drug resistance and its low nephrotoxicity. The optimal and safe dose for caspofungin in neonates with kidney impairment is yet to be established. IV fluconazole at a dose of 10 mg/kg/day was added because of persistent elevation of CRP levels. Reported MICs at 48 h for *C. parapsilosis* were 0.5 mg/L for 5-flucytosine, 2 mg/L for fluconazole, 0.125 mg/L for voriconazole and 1.5 mg/L for amphotericin B. Resolution of the episode, with the improvement of clinical conditions and urine output was reported after 10 days of treatment, and medications were gradually discontinued and followed by a two-week oral course of fluconazole (10 mg/kg/day). *C. parapsilosis* peritonitis is known to be more aggressive, with higher complication rates as compared to other *Candida* species.

##### Adolescents

Oualha et al. described fluconazole PK reporting the case of a 17-year-old patient requiring CVVHD and receiving IV fluconazole for *C. parapsilosis* cholecystits, with sensitivity to fluconazole (MIC 0.5 mg/L) (49). The administered dose was 10 mg/kg/day, that were continued until day 63, when the patient developed biliary fungal peritonitis and septic shock, leading to death. Drug concentrations were measured in plasma and dialysis effluent through a high-performance liquid chromatography assay with ultraviolet detection. An individual PK model was developed to calculate the cumulated drug amount recovered in the dialysis effluent, using the effluent flow rate (4,090 ml/h) and volumes at each time sampling. The estimated PK parameters (Table 10) were used to plot the plasma time profile of fluconazole concentrations, the plasma AUC curve and plasma trough concentrations. Fluconazole bile concentrations were estimated to be similar to those in plasma, suggesting an adequate bile excretion. Fluconazole elimination was high in bile and dialysis route, and higher than in healthy individuals, due to an absent tubular reabsorption in an anuric patient. The Authors failed to integrate also bile concentrations in the model, due to uncertain bile volumes and flows. The concentrations fell below the 10 mg/L target 10 h after the first dose. A loading dose of 900 mg with a maintenance dose of 600 mg twice daily were identified as the best regimen to reach optimal peak and trough concentrations.

As discussed by the Authors, higher loading doses and daily maintenance doses in 2 daily administrations may be suggested in individuals on CVVHD with candidiasis, especially in *Candida spp* with an MIC >2 mg/L. Due to the notable inter-individual and intra-individual variabilities, repeated therapeutic drug monitoring is a valuable option, especially in the infection site.

## Discussion

To our knowledge, this is the first Systematic Review aiming at gathering and examining the available evidence in the literature behind current dosing recommendations for the use of anti-infective drugs in pediatric patients with pre-existing kidney dysfunction, to guide in estimating drug doses for these populations. The critical aspects of drug dosing in children lie in establishing the need for adjustment according to variables such as age, weight vs. body surface area. In case of impaired kidney function, either CKD or AKI, molecule characteristics and reduced clearance should also be considered, even though it is hard to identify with certainty every relevant influence on drug action in every age subgroup, from newborns to adolescents. The main issue is drug accumulation, resulting in over-dosing. There is no universal rule, and sometimes no adjustment may be required from adult dosing. Dosing interval alone may be extended in case of long plasma half-lives, or dosing size may be reduced in case of a narrow therapeutic range (4).

The retrieved studies were mainly about molecules administered intravenously in ICU contexts, common in cases of severe infections in patients with pre-existing kidney dysfunction. Moreover, its is estimated that one in four children admitted to PICU will develop AKI and 10 to 15% will need CKRT, with different HD modalities (121). Antibiotic dosing in critically ill patients undergoing CKRT is challenging, with a risk of sub-therapeutic dosing, one of the main contributing factors to antibiotic resistance development and mortality (122, 123). This sums up the marked changes in PK during critical illness due to the presence of systemic inflammatory response syndrome, capillary leak syndrome, hypoalbuminemia, or dysregulation in renal blood flow (124). Critically ill children usually have a higher total body and extracellular water because of fluid overload, commonly present in these patients. This increase in total body and extracellular water can raise the apparent distribution volume at steady state (and consequently half-life) for water-soluble drugs.

Considering the neonatal sub-population, PD is of paramount importance in newborns and infants with hemodynamic instability or at risk of bleeding in case of AKI during serious, life-threatening infections or in case of paucity/failure of vascular access. It is technically more straightforward and avoids the need for vascular accesses and extracorporeal blood circuits. Peritonitis is one of the most common complications, which has led to the practice of intraperitoneal administration of anti-infective drugs.

Last, inter-individual variabilities must be considered in these complex contexts while defining the most appropriate dosing regimen, ensuring safety and efficacy with therapeutic drug monitoring and adjustments by the development of PK models based on Monte Carlo or Bayesian simulations, whenever possible (27, 28, 31, 42, 49).

### Limitations

The main limitation of this review relies on the heterogeneity of the included study types, mainly ranging from case reports to prospective/retrospective studies, that show reduced potency due to small patient samples, with only one RCT. In addition, almost all retrieved studies had an observational design, with a weaker methodological quality of reporting evidence than RCTs. For this reason, statistical analyses were not conducted.

### Conclusions and Final Implications for Clinical Practice

Dosing size or interval adjustments in pediatric patients with renal impairment vary according to age, critical illness status, degree of decreased renal function and dialysis type. This study offers to guide clinical practice with the available evidence for safe and effective dosing of anti-infective medicines in this special population. Dose adjustments should be guided not only by serum trough concentration monitoring alone but by Bayesian forecasting whenever possible, based on PK models. Our findings underline the relevance of population PK in clinical practice in special populations and highlight the urgent need of developing predictive specific models for critical pediatric patients.

## Data Availability Statement

The original contributions presented in the study are included in the article/Supplementary Material, further inquiries can be directed to the corresponding author.

## Author Contributions

CM, EB, and DDonà: conceptualization, methodology, and writing-review and editing. CM, EB, DDonà, and CG: validation. CM, DDoni, and CI: investigation and resources and data curation. CM: writing-original draft preparation. CG, DDonà, and EB: visualization and supervision. All the authors have approved the submitted version and agree to be personally accountable for the authors' own contributions and for ensuring that questions related to the accuracy or integrity of any part of the work, even ones in which the author was not personally involved, are appropriately investigated, resolved, and documented in the literature.

## Funding

This project was supported by the Residency program in Pediatrics of the Department of Women's and Children's Health - University of Padova.

## Conflict of Interest

The authors declare that the research was conducted in the absence of any commercial or financial relationships that could be construed as a potential conflict of interest.

## Publisher's Note

All claims expressed in this article are solely those of the authors and do not necessarily represent those of their affiliated organizations, or those of the publisher, the editors and the reviewers. Any product that may be evaluated in this article, or claim that may be made by its manufacturer, is not guaranteed or endorsed by the publisher.
