# Supplementary material for: Anti-infective Medicines Use in Children and Neonates With Pre-existing Kidney Dysfunction: A Systematic Review
Source: Front Pediatr. 2022 Apr 26;10:868513. doi: 10.3389/fped.2022.868513 (PMC9087830; doi:10.3389/fped.2022.868513)
Supplement: Supplementary file 1 [file Data_Sheet_1.pdf]

Full search strategy

Embase-Ovid Medline (1410 retrieved papers); Cochrane (382 retrieved papers)

Embase 1974 to 2021 Week 35, Ovid MEDLINE(R) ALL 1946 to September 21, 2021

1. (newborn* or neonat* or infan* or toddler* or pre-schooler* or preschooler* or child* or children or adolescen* or pediatr* or paediatr* or youth* or teen or teens or teenage* or kid or kids or baby or babies).mp. [mp=ti, ab, hw, tn, ot, dm, mf, dv, kw, fx, dq, nm, kf, ox, px, rx, an, ui, sy]

AND

2. (anti-bacterial* or antibacterial* or anti-mycobacterial* or antimycobacterial* or antibiotic* or antiviral* or antifungal* or antimalaric* or antiprotozoal* or antielminthic* or antiparasitic*).mp. [mp=ti, ab, hw, tn, ot, dm, mf, dv, kw, fx, dq, nm, kf, ox, px, rx, an, ui, sy]

AND

3. (dosing* or dosage* or dose*).mp. [mp=ti, ab, hw, tn, ot, dm, mf, dv, kw, fx, dq, nm, kf, ox, px, rx, an, ui, sy]

AND

4. (Renal impairment* or renal dysfunction* or renal failure* or acute kidney injury* or chronic kidney disease* or end-stage renal disease*).mp. [mp=ti, ab, hw, tn, ot, dm, mf, dv, kw, fx, dq, nm, kf, ox, px, rx, an, ui, sy]

5. 1 and 2 and 3 and 4

Figure Supplementary 1: Risk of bias assessment for Clinical Trials

		Schaefer et al, 1999
Selection bias	Random sequence generation	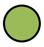
	Allocation concealment	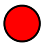
Performance bias	Blinding of participants and personnel	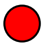
Detection bias	Blinding of outcome assessment	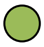
Attrition bias	Incomplete outcome data	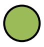
Reporting bias	Selective reporting	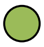
Other bias	Anything else, ideally prespecified	/
**Overall bias**		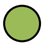

Figure Supplementary 2.1: Risk of bias assessment for observational studies (example for retrospective study)

Criteria	Yes	No	Other (CD, NR, NA)*
1. Was the research question or objective in this paper clearly stated?	X		
2. Was the study population clearly specified and defined?	X		
3. Was the participation rate of eligible persons at least 50%?			NA
4. Were all the subjects selected or recruited from the same or similar populations (including the same time period)? Were inclusion and exclusion criteria for being in the study prespecified and applied uniformly to all participants?		X	
5. Was a sample size justification, power description, or variance and effect estimates provided?		X	
6. For the analyses in this paper, were the exposure(s) of interest measured prior to the outcome(s) being measured?	X		
7. Was the timeframe sufficient so that one could reasonably expect to see an association between exposure and outcome if it existed?	X		
8. For exposures that can vary in amount or level, did the study examine different levels of the exposure as related to the outcome (e.g., categories of exposure, or exposure measured as continuous variable)?		X	
9. Were the exposure measures (independent variables) clearly defined, valid, reliable, and implemented consistently across all study participants?		X	
10. Was the exposure(s) assessed more than once over time?	X		
11. Were the outcome measures (dependent variables) clearly defined, valid, reliable, and implemented consistently across all study participants?		X	NA
12. Were the outcome assessors blinded to the exposure status of participants?		X	
13. Was loss to follow-up after baseline 20% or less?			NA
14. Were key potential confounding variables measured and adjusted statistically for their impact on the relationship between exposure(s) and outcome(s)?		X	

Figure Supplementary 2.2: Risk of bias assessment for observational studies

Study	1.	2.	3.	4.	5.	6.	7.	8.	9.	10.	11.	12.	13.	14.	rating
**Cies et al, USA, 2016 [21]**	*y*	*y*	*NA*	*y*	*n*	*y*	*y*	*y*	*y*	*y*	*n*	*n*	*NA*	*y*	*Fair*
**Company-Albir et al, Spain, 2019 [22]**	*y*	*y*	*NA*	*NA*	*n*	*y*	*y*	*n*	*n*	*n*	*n*	*n*	*NA*	*n*	*Poor*
**Fitzgerald et al, USA, 20 19 [23]**	*y*	*y*	*NA*	*y*	*n*	*y*	*y*	*y*	*y*	*y*	*n*	*n*	*NA*	*y*	*Fair*
**Sridharan et al, Bahrain, 2019 [24]**	*y*	*y*	*NA*	*y*	*n*	*y*	*y*	*y*	*y*	*y*	*n*	*n*	*NA*	*y*	*Fair*
**Sridharan et al, Bahrain, 2019 [25]**	*y*	*y*	*NA*	*y*	*n*	*y*	*y*	*y*	*y*	*y*	*n*	*n*	*NA*	*y*	*Fair*
**Abid et al, USA, 2020 [26]**	*y*	*y*	*NA*	*NA*	*n*	*y*	*y*	*n*	*n*	*n*	*n*	*n*	*NA*	*n*	*Poor*
**Smit et al, USA, 2021 [27]**	*y*	*y*	*y*	*y*	*y*	*y*	*y*	*y*	*y*	*y*	*y*	*n*	*n*	*y*	*Good*
**Goldstein et al, USA, 2001 [28]**	*y*	*y*	*y*	*y*	*n*	*y*	*y*	*y*	*y*	*y*	*n*	*n*	*NA*	*y*	*Fair*
**Alqaqaa et al, USA, 2016 [29]**	*y*	*y*	*NA*	*NA*	*n*	*y*	*y*	*n*	*n*	*n*	*n*	*n*	*NA*	*n*	*Poor*
**Cies et al, USA, 2016 [30]**	*y*	*y*	*NA*	*NA*	*n*	*y*	*y*	*n*	*n*	*n*	*n*	*n*	*NA*	*n*	*Poor*
**Rapp et al, France, 2020 [31]**	*y*	*y*	*y*	*y*	*n*	*y*	*y*	*y*	*y*	*y*	*n*	*n*	*NA*	*y*	*Fair*
**Hayakawa et al, Japan, 2006 [32]**	*y*	*y*	*y*	*y*	*n*	*y*	*y*	*y*	*y*	*y*	*n*	*n*	*NA*	*y*	*Fair*

**Shetty et al, USA, 2011 [33]**	*y*	*y*	*NA*	*NA*	*n*	*y*	*y*	*n*	*n*	*n*	*n*	*n*	*NA*	*n*	*Poor*
**Cies et al, USA, 2015 [34]**	*y*	*y*	*NA*	*NA*	*n*	*y*	*y*	*n*	*n*	*n*	*n*	*n*	*NA*	*n*	*Poor*
**Ozsurekci et al, Turkey, 2021 [35]**	*y*	*y*	*y*	*y*	*n*	*y*	*y*	*y*	*y*	*y*	*n*	*n*	*NA*	*y*	*Fair*
**Yoshioka et al, Japan, 1978 [36]**	*y*	*y*	*y*	*y*	*n*	*y*	*y*	*y*	*y*	*y*	*n*	*n*	*NA*	*y*	*Fair*
**Sirinavin et al, USA, 1980 [37]**	*y*	*y*	*y*	*y*	*n*	*y*	*y*	*y*	*y*	*y*	*n*	*n*	*NA*	*y*	*Fair*
**Lanao et al, Spain, 1981 [38]**	*y*	*y*	*y*	*y*	*n*	*y*	*y*	*y*	*y*	*y*	*n*	*n*	*NA*	*y*	*Fair*
**Shetty et al. USA,2005 [40]**	*y*	*y*	*NA*	*NA*	*n*	*y*	*y*	*n*	*n*	*n*	*n*	*n*	*NA*	*n*	*Poor*
**Stitt et al, USA, 2019 [41]**	*y*	*y*	*NA*	*y*	*n*	*y*	*y*	*y*	*y*	*y*	*n*	*n*	*NA*	*y*	*Fair*
**Butragueño-Laiseca et al, Spain, 2020 [42]**	*y*	*y*	*y*	*y*	*n*	*y*	*y*	*y*	*y*	*y*	*n*	*n*	*NA*	*y*	*Fair*
**Chan et al, USA, 2012 [43]**	*y*	*y*	*NA*	*NA*	*n*	*y*	*y*	*n*	*n*	*n*	*n*	*n*	*NA*	*n*	*Poor*
**Morris et al. UK, 2017 [44]**	*y*	*y*	*NA*	*NA*	*n*	*y*	*y*	*n*	*n*	*n*	*n*	*n*	*NA*	*n*	*Poor*
**Linder et al, Israel, 2003 [45]**	*y*	*y*	*y*	*y*	*n*	*y*	*y*	*y*	*y*	*y*	*n*	*n*	*NA*	*y*	*Fair*
**Cheng et al, Taiwan 2010 [46]**	*y*	*y*	*NA*	*NA*	*n*	*y*	*y*	*n*	*n*	*n*	*n*	*n*	*NA*	*n*	*Poor*
**Dumangin et al, France, 2020 [47]**	*y*	*y*	*NA*	*NA*	*n*	*y*	*y*	*n*	*n*	*n*	*n*	*n*	*NA*	*n*	*Poor*
**Healy et al, USA, 2011 [48]**	*y*	*y*	*NA*	*NA*	*n*	*y*	*y*	*n*	*n*	*n*	*n*	*n*	*NA*	*n*	*Poor*
**Oualha et al, France, 2019 [49]**	*y*	*y*	*NA*	*NA*	*NA*	*NA*	*y*	*n*	*n*	*n*	*NA*	*n*	*n*	*n*	*Poor*

Legends: *y: yes; n:no; NA: not applicable*
